# High‐Speed Atomic Force Microscopy Reveals the Dynamic Interplay of Membrane Proteins is Lipid‐Modulated

**DOI:** 10.1002/smsc.202500258

**Published:** 2025-07-08

**Authors:** Eunji Shin, Yining Jiang, Batiste Thienpont, James N Sturgis, Simon Scheuring

**Affiliations:** ^1^ Department of Anesthesiology Weill Cornell Medicine 1300 York Avenue New York NY 10065 USA; ^2^ Laboratoire d’Ingénierie des Systèmes Macromoléculaires (LISM) Unité Mixte de Recherche (UMR) 7255 Centre National de la Recherche Scientifique (CNRS) Aix Marseille Université 13402 Marseille France; ^3^ Department of Physiology and Biophysics Weill Cornell Medicine 1300 York Avenue New York NY 10065 USA

**Keywords:** high‐speed atomic force spectroscopy, membrane diffusion, membrane proteins, protein dynamics, reconstitutions

## Abstract

The solvent of membrane proteins is the membrane lipids in which they are embedded. Therefore, the nature of the lipids that surround membrane proteins impacts their dynamics and interactions. Unfortunately, how membrane proteins dynamically interact is difficult to study, and little is experimentally known how membrane proteins interplay in a membrane at the molecular scale. Herein, high‐speed atomic force microscopy (HS‐AFM) is used to dynamically image a well‐controlled bottom‐up system consisting of two aquaporin‐fold membrane proteins, pentameric FocA and tetrameric GlpF, that interact in membranes composed of varying amounts of 1,2‐dioleoyl‐sn‐glycero‐3‐phosphocholine (DOPC) and *E. coli* lipids. It is found that the lipid environment significantly influences membrane protein mobility and interaction, where increased *E. coli* lipid content reduces protein movement, while DOPC‐rich environments promote mobility. Furthermore, the supramolecular structures of the membrane proteins and protomer interactions in clusters are also lipid modulated, where *E. coli* lipids favor specific protein–protein interactions, whereas greater interaction variability is found in DOPC. These findings highlight the role of lipids in regulating protein diffusion and interactions and suggest that lipid–protein interaction energetics play a significant role in controlling membrane protein interactions and supramolecular assembly.

## Graphical abstract

High‐speed atomic force microscopy captures the lipid‐dependent clustering dynamics of pentameric FocA and tetrameric GlpF in supported lipid bilayers. Protein mobility, cluster stability, and packing geometry vary with lipid composition, revealing how membrane environments regulate supramolecular membrane protein organization. A model is proposed in which lipid‐driven interactions govern cluster formation, providing insights into membrane protein organization at the mesoscale.

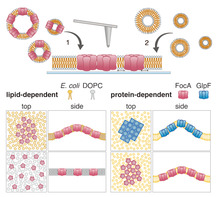

## 1 Introduction

Membrane proteins constitute ≈25% of the proteome in *Escherichia coli* and up to 30% in *Homo sapiens*.^[^ ^1^ ^]^ They are amphiphilic in nature, interacting with both aqueous and lipid bilayer environments. Transmembrane proteins span the entire hydrophobic thickness of the lipid bilayer, and are exposed on their faces to the aqueous cytoplasmic and extracellular or organellar solution, respectively.^[^ ^2^, ^3^ ^]^ Membrane proteins perform various physiological functions in the cell membrane, such as transport and signaling, and their activity is modulated by the surrounding lipid membrane environment.^[^ ^4^, ^5^ ^]^ The structure, dynamics, and function of membrane proteins can be regulated by specific interactions with lipids,^[^ ^6^ ^]^ making the study of membrane protein–lipid interactions essential for understanding the functional mechanisms of membrane proteins.^[^ ^7^, ^8^ ^]^

Biological membranes are primarily composed of phospholipids, protein and sugars. The phospholipids are complex mixtures of many different lipid species, in terms of lipid headgroup and tail length and/or saturation, which, in addition, vary depending on their location. In eukaryotes, transmembrane proteins are transported through the secretory pathway, from the endoplasmic reticulum (ER) through the Golgi apparatus to the plasma membrane (PM). During this process, the membrane composition changes: The contents of saturated lipids increase while unsaturated lipids decrease, and concentrations of cholesterol, anionic lipids, and sphingolipids rise. Consequently, as membrane proteins progress through the secretory pathway, they encounter a thicker, more densely packed, more rigid, and more viscous lipid environment.^[^ ^3^, ^9^, ^10^ ^]^ Variations in lipid types, concentrations, membrane thickness, and fluidity can impact protein structure, function, and propensity to interact and form supramolecular structures. Thus, studying the interplay between lipids and membrane proteins is crucial for understanding these regulatory mechanisms at the molecular level.

With advances in our understanding of how lipids regulate protein function, high‐resolution structural analysis of membrane proteins using cryogenic electron microscopy (cryo‐EM) has expanded our knowledge of membrane protein–lipid interactions.^[^ ^6^, ^11^, ^12^ ^]^ In addition to structures, atomic‐level information from simulations and native mass spectrometry inform about the interactions between lipid molecules and membrane proteins, highlighting the role of the lipid environment in these interactions.^[^ ^13^, ^14^ ^]^ However, since cell membranes are liquid‐crystalline assemblies of lipids and proteins that are dynamic,^[^ ^15^ ^]^ it is essential to study these interactions in conditions that mimic the biological contexts, i.e., membrane proteins in a continuous membrane in solution and at ambient temperature and pressure, and to observe the system at high spatial and temporal resolution.

Here, we used high‐speed atomic force microscopy (HS‐AFM) to observe the lipid‐dependent interaction between membrane proteins in real time.^[^ ^16^ ^]^ HS‐AFM features miniaturized cantilevers and high‐frequency response feedback control systems, allowing them to surpass the frame acquisition rate of conventional AFM, and record images at sub‐second time scale for real‐time observation of dynamic molecular changes.^[^ ^17^ ^]^ In biological applications, particularly in membrane protein research, HS‐AFM has proven a vital tool for studying protein–ligand interactions,^[^ ^18^ ^]^ protein–protein interactions,^[^ ^19^ ^]^ and conformational changes.^[^ ^20^, ^21^, ^22^ ^]^

Here, to investigate how the lipid environment modulates membrane protein interactions and dynamics, we built a controlled minimal component bottom‐up system, allowing us to study the behavior of two *E. coli* membrane proteins, pentameric FocA and tetrameric GlpF, in *E. coli* lipid environments. We found that the mobility and the arrangement of membrane proteins within protein clusters were substantially modulated by changes in the lipid environment. Detailed interaction analysis of the clustered membrane proteins revealed that the annular lipid composition modulated how membrane proteins interact with each other, where the possibility to recruit specific lipids from the heterogeneous *E. coli* mixture enhances interaction energies and favors tight interactions. Finally, we mixed FocA and GlpF and explored how the internal symmetry of the oligomeric membrane proteins influences their interactions.

## 2 Results

### 2.1 Lipid‐Dependent Interaction Dynamics of Pentameric FocA

**Figure 1:**
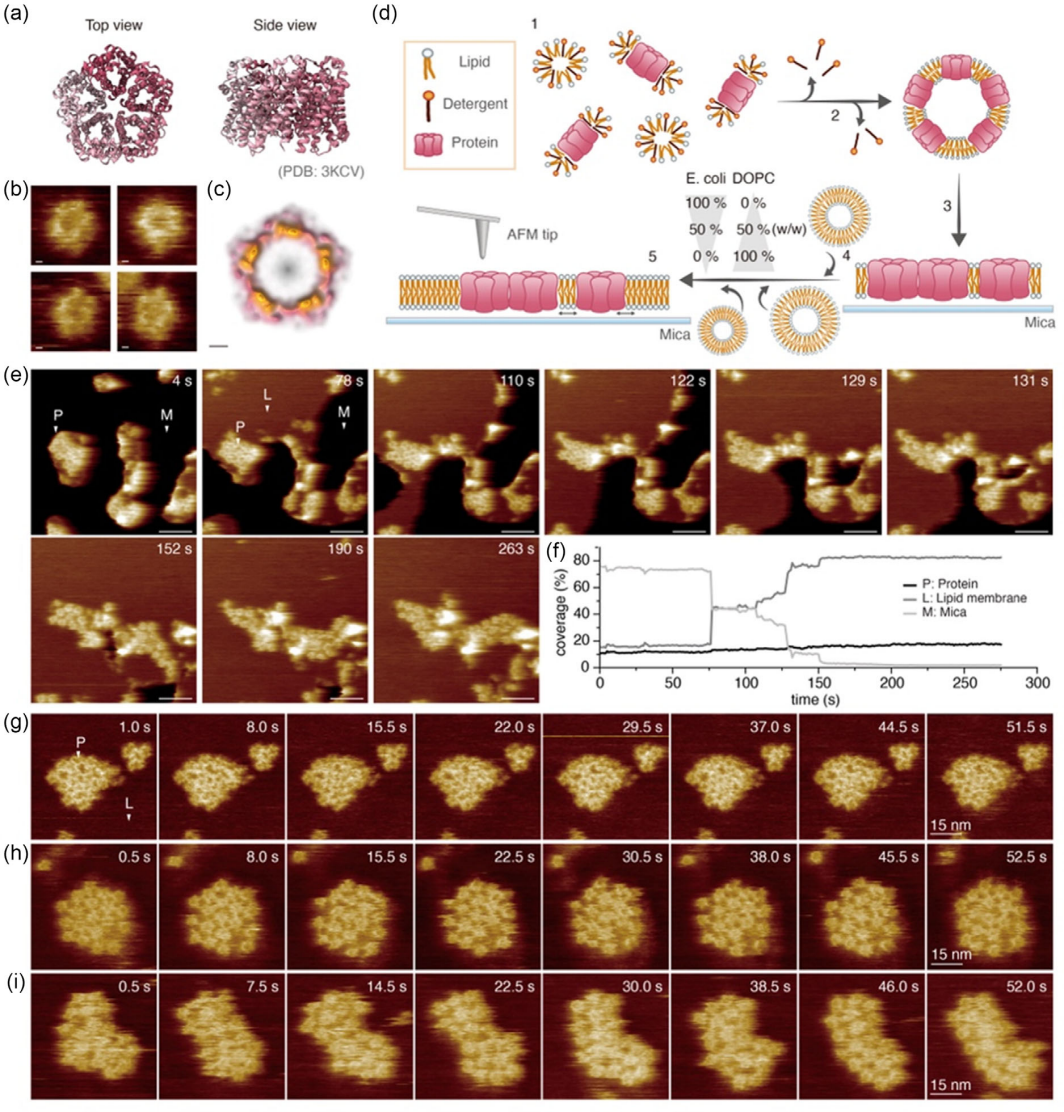
FocA structure and experimental design to study lipid‐dependent protein–protein interactions with HS‐AFM. a) Top (left) and side (right) views of FocA (PDB 3KCV). b) HS‐AFM images of four individual FocA pentamers viewed from the extracellular side. Scale bars: 1 nm. c) LAFM map of FocA. Scale bar: 1 nm. d) Schematic of the experimental design: Step 1: Detergent‐solubilized FocA and lipid are mixed. Step 2: Detergent removal through dialysis. Step 3: Resulting proteo‐liposomes are adsorbed to freshly cleaved, atomically flat mica. Step 4: Empty lipid vesicles of controlled lipid composition are added to the sample surface and allowed to form a continuous SLB. e) HS‐AFM movie frames showing the lipid membrane fusion process to FocA clusters on mica. Here, 85% *E. coli* lipid and 15% DOPC lipid (w/w) (*χ* = 0.85). P: Protein (FocA), M: Mica, L: lipid membrane. Scale bar: 25 nm. f) Surface coverage analysis of (e). The graph displays the areas occupied by FocA (P, black), lipid membrane (L, dark gray), and mica (M, light gray). g–i) HS‐AFM movie frames of individual FocA cluster dynamics. Movies were acquired at an imaging rate of 2 frames per second and a scan area of 80 × 80 nm. Lipid conditions: 100% *E. coli* lipids (*χ* = 0.0) g), 75% DOPC/25% *E. coli* lipid (*χ* = 0.75) h), and 85% DOPC/15% *E. coli* lipid (*χ* = 0.85) i).

The prokaryotic formate transporter, FocA, plays a vital role in facilitating the movement of formate across the membrane. Despite of its name, FocA is a passive channel rather than an active transporter enabling the uptake or release of formate as required by the cell.^[^ ^23^ ^]^ Structurally, FocA is a pentamer with each protomer composed of six transmembrane α‐helices (**Figure** **1**a). Two loops, each folding into a short helix, enter the central protomer region from both sides and form together a substrate selectivity filter. Thus, the six transmembrane helices form roughly an hour‐glass‐shaped channel where the entrant loops create the formate‐selective channel.^[^ ^23^, ^24^, ^25^ ^]^ Despite minimal sequence homology, the protomer structure shares many similarities with aquaporins (AQPs), where also six helixes surround two re‐entrant loops that convey water selectivity to the channels.^[^ ^26^ ^]^

To image FocA in a continuous lipid membrane using HS‐AFM, we employed the membrane extension membrane protein reconstitution (MEMPR, Experimental Section) method.^[^ ^19^, ^27^ ^]^ HS‐AFM allowed to image individual isolated FocA molecules, faithfully reporting their pentameric architecture (Figure 1b and Movie S1, Supporting Information). Time‐averaged HS‐AFM images of individual FocA molecules revealed polar plot profiles with five protrusions peaks of ≈72° periodicity, allowing for computational, unbiased protein classification (Figure S1a, Supporting Information). This observation corroborated that FocA formed a stable pentamer also within a continuous lipid membrane, consistent with previous structural characterizations of isolated molecules.^[^ ^23^, ^24^, ^25^ ^]^ Unlike *E.coli* aquaporin‐Z (AqpZ) that reconstituted with up‐and‐down orientation in the membrane,^[^ ^19^ ^]^ FocA molecules were uniformly inserted into the membrane, exposing only the extracellular face in HS‐AFM experiments, thus presenting a native‐like topology (Discussion). From these HS‐AFM images (Figure 1b) we calculated a 3D localization AFM (3D‐LAFM)^[^ ^28^, ^29^ ^]^ map of FocA that resolved two major and two minor topographic features per subunit (Figure 1c). Structural comparison of the 3D‐LAFM map with the atomic model (PDB 3KCV)^[^ ^30^ ^]^ suggested that HS‐AFM observed exclusively the extracellular surface of FocA.

To investigate how lipids modulate FocA protein–protein interactions at the single molecule level, we designed the following experimental procedure (Figure 1d): First, the detergent‐solubilized FocA was mixed with detergent‐solubilized lipids (Figure 1d, step 1, Experimental Section) and reconstituted through detergent removal via dialysis, resulting in the formation of proteo‐liposomes (Figure 1d, step 2). The lipid‐to‐protein ratio (LPR) was set very low, at 0.2–0.3 (w:w), to minimize the lipid content at this stage (Experimental Section). The reconstituted proteo‐liposomes were then absorbed onto an atomically flat mica surface (Figure 1d, step 3). Subsequently, vesicles of controlled lipid content were added to the sample chamber (Figure 1d, step 4), leading to vesicle adsorption and bilayer spreading on the mica, followed by bilayer fusion with the previously adsorbed and spread proteo‐liposomes. This finally led to the formation of a continuous supported lipid bilayer (SLB) with islands of clustered FocA (Figure 1d, step 5). To explore the effect of the lipid environment on membrane protein interactions and dynamics, the addition of various lipid compositions can be controlled in step 4 of the procedure. Specifically, we supplemented FocA with mixtures of *E. coli* total lipid and 1,2‐dioleoyl‐sn‐glycero‐3‐phosphocholine (DOPC) lipid at different ratios, *χ*, where *χ* denotes the fraction of DOPC to total lipids (*χ* = DOPC/(DOPC +* E. coli*)) (w/w). This experimental design allows us to relate changes in lipid environment to the behavior and interaction of FocA within the membrane.

The SLB formation process was recorded in real time at an imaging rate of 1 frame per second (Movie S2, Supporting Information). Representative HS‐AFM movie frames show the process of lipid membrane fusion with the proteo‐liposomes (Figure 1e). The analysis of the progression of the SLB surface coverage over time (Figure 1f) showed that initially FocA molecules covered ≈11% (Figure 1e, *t* = 4 s, arrowhead *P*, Figure 1f), bare mica ≈74% (Figure 1e, *t* = 4 s, arrowhead *M*, Figure 1f), while lipid covered ≈15% (Figure 1e, *t* = 4 s, Figure 1f) of the imaging area. Upon the addition of empty vesicles to the protein clusters, membrane fusion occurred, leading to the formation of an SLB covering the mica (Figure 1e, *t* = 78 s, arrowhead *L*, Figure 1f). ≈75 s after the onset of lipid fusion, the membrane completely covered the mica surface within the scan area, forming a continuous SLB with embedded FocA clusters (Figure 1e, *t* = 263 s, Figure 1f). Subsequent observations revealed that protein clusters remained overall stable within the lipid environment, but featured dynamics of protein associations, dissociations, and rearrangements (Movie S2, Supporting Information).

After the SLB formation process was complete, we monitored the dynamics of individual protein clusters (Movie S3, Supporting Information). The dynamic behavior of the FocA proteins in clusters showed variations depending on the lipid environment. Overall, a noticeable trend emerged: A decrease in the fraction of *E. coli* lipids correlated with an increase in the protein mobility in the observed clusters (Movie S3, Supporting Information). In cases where 100% *E. coli* lipids (*χ* = 0.0) were introduced, the FocA clusters exhibited limited movement (Figure 1g and Movie S3, Supporting Information). Whereas, in a 25% *E. coli* lipid (*χ* = 0.75) environment, a slightly increased mobility was observed (Figure 1h, Movie S3, Supporting Information), and in a 15% *E. coli* lipid (*χ* = 0.85), the clusters exhibited pronounced dynamics (Figure 1i and Movie S3, Supporting Information). Interestingly, in 0% *E. coli* lipid (*χ* = 0.0) environment, FocA protein clusters dissociated after the addition of lipids (Figure S2a, Supporting Information).

### 2.2 Protein Cluster Dynamics and Energetics in Varying Lipid Compositions

**Figure 2:**
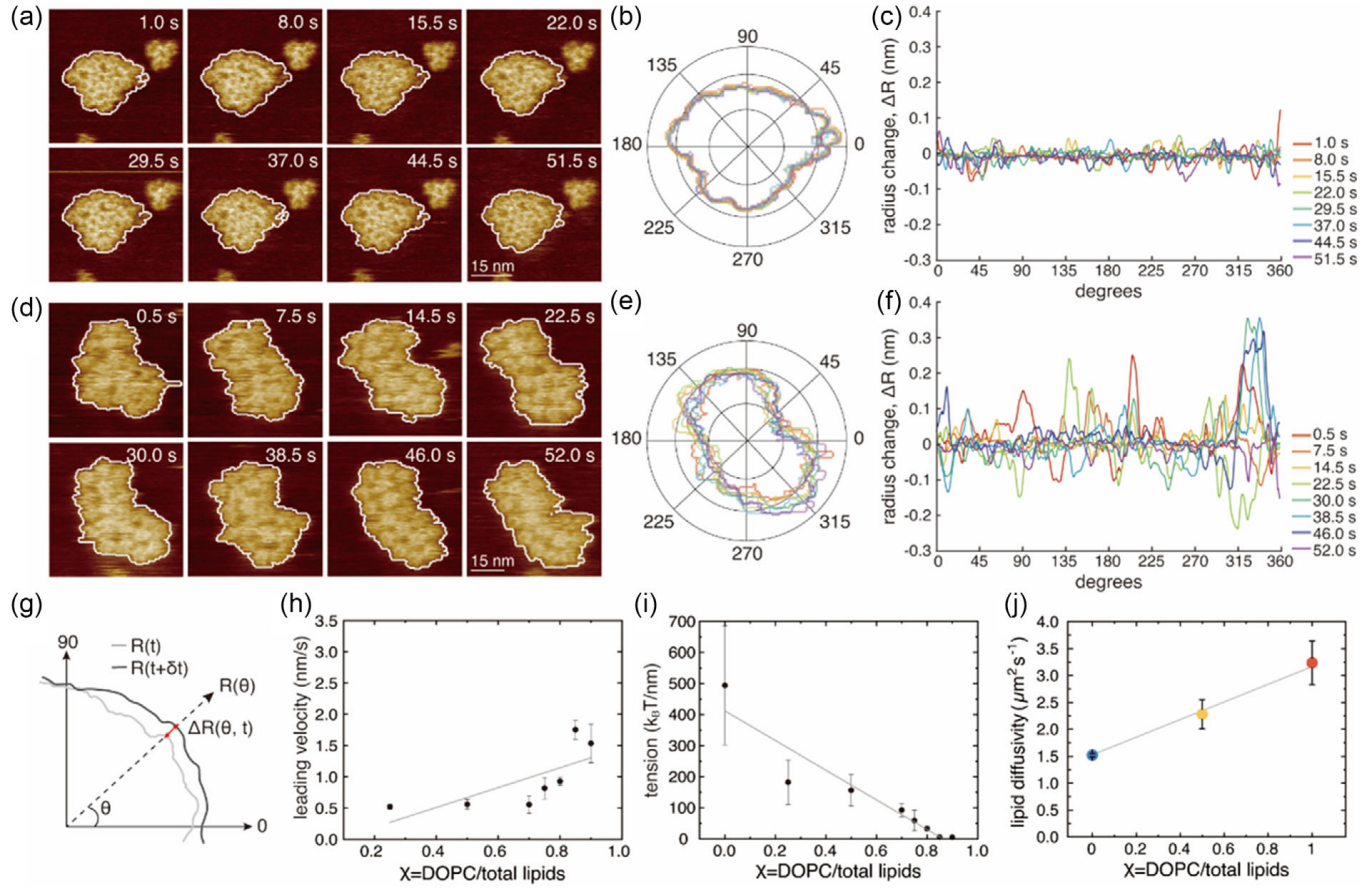
FocA cluster dynamics and interaction energetics are lipid dependent. a–c) FocA in 100% *E. coli* lipid (*χ* = 0.0). d–f) FocA in 15% *E. coli* lipid (*χ* = 0.85). a,d) Representative contours identifying protein cluster boundaries in HS‐AFM movie frames, (b,e) representative cluster contours in polar coordinates, and (c,f) radius change, Δ*R*, as a function of angle. Recording times of colored traces displayed in (b,c,e,f) are indicated on the right. g) Illustration of the determination of radius change, Δ*R*, i.e., the difference in radial distance from cluster center of mass at angle (*θ*) between consecutive frames (*t, t +* δt). h) Mean leading‐edge velocity ($\mu_{v}$, nm s^−1^) as a function of DOPC lipid to total lipid ratio (*χ* = DOPC/total lipids) ranging from 0 to 1. Line illustrates the trend of increasing mean leading‐edge velocity with increasing *χ* (decreasing *E. coli* lipid ratio). i) Line tension, *λ*, *k*_B_T nm^−1^, as a function of DOPC/total lipids ratio *χ*. Line illustrates the trend of decreasing line tension with increasing *χ*. Data points in h,i) are derived from at least five protein clusters at each lipid condition (mean and standard deviation, marker, and error bar). j) Diffusion coefficients of membranes constituted of *E. coli*/DOPC/NBD‐PC = 98/0/2 (blue), 50/48/2 (yellow), and 0/98/2 (red). Marker and error bars are mean and standard deviation values from three or more different FRAP areas (Figure S3, Supporting Information). Line illustrates the trend of increasing lipid diffusivity with increasing DOPC content.

To analyze the molecular dynamics of protein assemblies, we first delineated the boundaries of the protein clusters (Experimental Section). To avoid errors that may arise from boundary delineation, we analyzed only clusters that were isolated from the beginning of the observation to the observation endpoint, excluding cases where multiple clusters fused or grouped together (under‐segmentation), or an individual cluster that divided into multiple entities (over‐segmentation).^[^ ^31^ ^]^ Each contour obtained from each frame acquired over time exhibits a distinct shape (**Figure** **2**a,d). Thus, to capture the spatial arrangement and evolution of protein clusters, we visualized the cluster boundary radial distance as a function of the polar coordinate (Figure 2b,e). Notably, due to the generally circular morphology of the contours, the use of polar coordinates centered on the center of mass of the protein cluster proved appropriate and intuitive.^[^ ^30^ ^]^ We used this approach to compare the structural variability of clusters in 100% (*χ* = 0.0, Figure 2a,b) and 15% (*χ* = 0.85, Figure 2d,e) *E. coli* lipid content. Each illustrated contour was derived from a comparable timeline (Figure 2b,e). Notably, at high *E. coli* lipid concentrations, minimal contour shape fluctuations were observed, while in a lipid environment dominated by DOPC with low *E. coli* lipid content, we observed pronounced contour morphology changes.

To quantify changes in cluster contour shape as a function of the lipid environment, we next calculated the radial distance changes of cluster boundaries between consecutive frames (*t, t+* δt) (Figure 2c,f), termed radius change, Δ*R* (Figure 2g, Equation (1).

(1) ΔR(θ,t)=R(θ,t+δt)−R(θ,t)

Positive radius change, Δ*R*, values at specific angles (*θ*) signify an extension, i.e., increase of the distance to the cluster edge from the center of mass, whereas negative values denote retraction at the given angular direction. Upon plotting Δ*R* by *θ*, we observed greater variability in 15% *E. coli* lipid content (*χ* = 0.85, Figure 2f) than in membranes composed of *E. coli* lipid only (*χ* = 0.0, Figure 2c). Subsequently, we derived the mean leading‐edge velocity ($\mu_{v},$ nm s^−1^, Experimental Section),^[^ ^30^, ^31^ ^]^ enabling us to quantify the trend of FocA cluster stability as a function of *χ*. We noted a decrease in $\mu_{v}$ with increasing *E. coli* lipid content (Figure 2h).

To retrieve an energy term characterizing the protein cluster stability within the lipid membrane, we analyzed the line tension, *λ* (*k*_B_T* nm* ^−1^), at the cluster circumference.^[^ ^32^, ^33^, ^34^ ^]^ We derived *λ* using boundary fluctuation analysis (Experimental Section),^[^ ^35^, ^36^ ^]^ allowing us to quantify and compare the morphological fluctuations of the protein clusters across different lipid compositions. Higher line tension correlates to increased cluster interaction energy. We observed a substantial increase in line tension with an increase in the proportion of *E. coli* lipids (Figure 2i). Starting with an average line tension value of 494 ± 191 *k*_B_T nm^−1^ in pure *E. coli* lipids (*χ* = 0), the line tension decreased as the fraction of *E. coli* lipid decreased, reaching 5.6 ± 1.1 *k*_B_T nm^−1^ in an environment where the *E. coli* lipid fraction was only 10% (*χ* = 0.90) (Figure 2i). As a reminder, in 0% *E. coli* lipids (*χ* = 0.0) FocA clusters dissociated (Figure S2a, Supporting Information). Thus, FocA clusters were stabilized by the availability of native lipids. Our results manifest how the lipid composition modulates protein–protein interaction energetics in protein clusters, and how the possibility of recruitment of native lipids from the *E. coli* lipid mixture favors the attractive interactions of the *E. coli* membrane protein FocA.

To gain further insight into the membrane characteristics, we conducted fluorescence recovery after photobleaching (FRAP) experiments. FRAP reveals large‐scale membrane fluidity, i.e., how swiftly fluorescent lipids repopulate a previously bleached membrane area, from which an average diffusion coefficient can be calculated^[^ ^37^ ^]^: We monitored FRAP on SLBs constituted of *E. coli*/DOPC/1‐Oleoyl‐2‐[12‐[(7‐nitro‐2‐1,3‐benzoxadiazol‐4‐yl)amino]dodecanoyl]‐sn‐Glycero‐3‐Phosphocholine (NBD‐PC) = 98/0/2, 50/48/2, and 0/98/2 (Figure S3a–c, Supporting Information) mixtures, giving diffusion coefficients (Figure S3d,e, Supporting Information, Experimental Section) of 3.2 ± 0.4, 2.3 ± 0.3, and 1.5 ± 0.03 μm^−2^ s^−1^ for 0, 50, and 98% *E. coli* lipid content, respectively (Figure 2j **)**. These results show that with increasing proportion of *E. coli* lipid membrane fluidity decreases, correlating with a reduced percentage of unsaturated acyl chains. Although FRAP and HS‐AFM experiments were performed on different substrates and using pure lipid and proteo‐liposomes, respectively, their qualitative agreement supports the results that lipid composition modulates membrane protein dynamics.

### 2.3 FocA–FocA Interactions are Lipid‐Dependent

**Figure 3:**
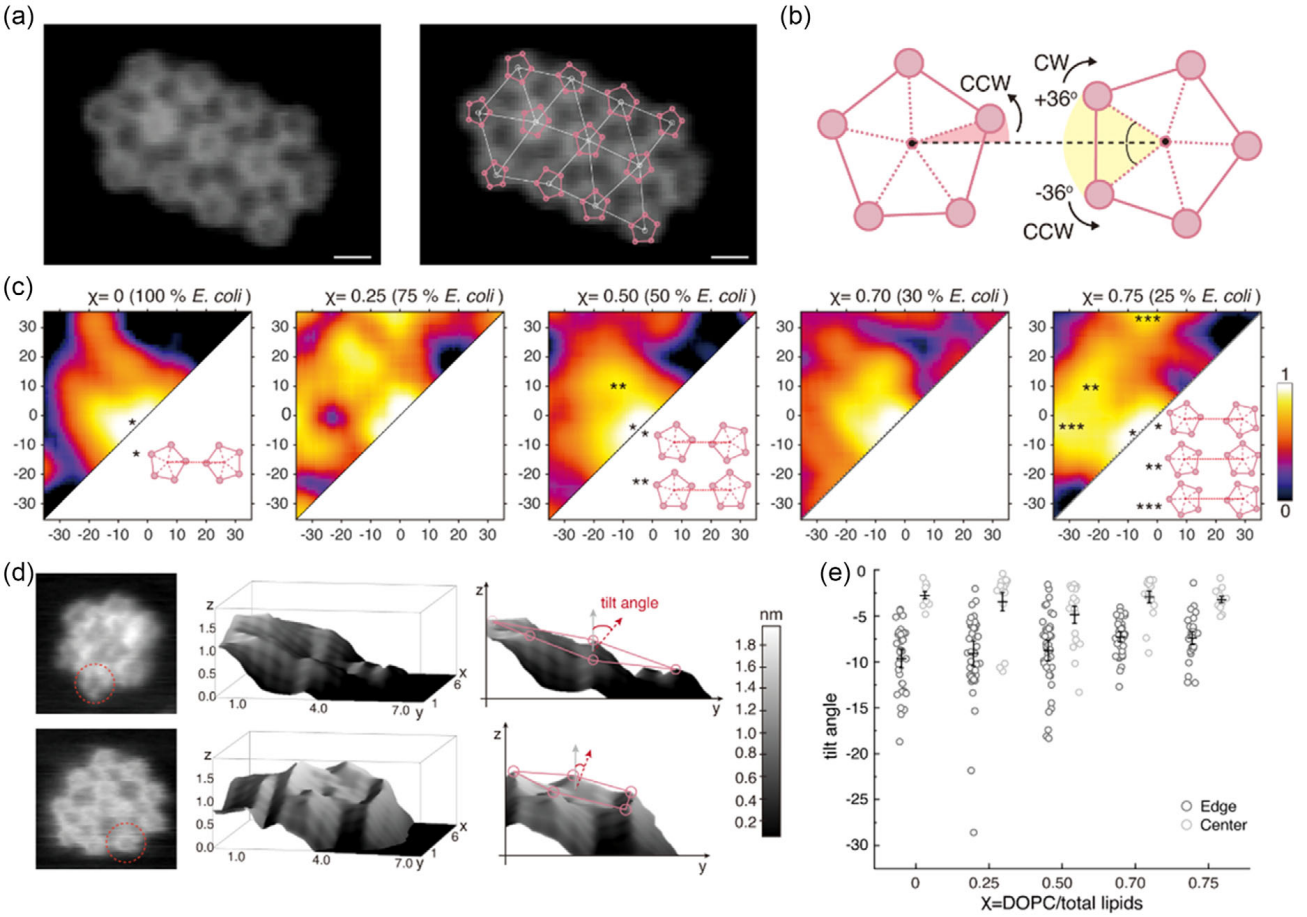
FocA–FocA molecular interactions are lipid modulated. a) Time‐averaged HS‐AFM frame (left) used for characterization of protein–protein interactions in FocA clusters (right): FocA pentamer centers (white dots) and protomers (pink dots). FocA pentamers were identified by connecting the five protomers, pink lines. White lines represent connections between FocA–FocA nearest neighbor pairs. Scale bar: 5 nm. b) Illustration depicting the interaction between adjacent protomers of neighboring FocA. The FocA–FocA neighbor connection (black dashed line) serves as angular reference to determine the angular location of the protomer closest to the neighbor FocA (shaded red in left molecule) where CCW and CW located protomers are denoted with negative and positive signs, respectively, within the range −36°–+36° (shaded yellow in right molecule). c) Probability density maps of interaction angles in various lipid environments. False‐color scale representing probability ranging from 0 to 1. Lipid conditions are denoted at the top of each map. The *x*‐ and *y*‐axes represent interaction angle ranges, −36°–+36°. Schematics of FocA interactions at angles indicated by the asterisks in panels *χ* = 0.0, 0.50, and 0.75. d) Tilt analysis of FocA in clusters in 100% (*χ* = 0.0, top) and 20% (*χ* = 0.80, bottom) *E. coli* lipid. Left: Representative HS‐AFM movie frames. A tilted FocA is indicated by a red circle. Center: 3D representation of tilted FocA from left image. Right: Illustration of FocA tilt angle analysis. Tilt angles were determined by the axis orthogonal to the plane defined by the five FocA protomers and the *z*‐plane. e) Tilt angle distributions as a function of lipid condition, see *χ* values. Data points in dark gray are tilt angles of molecules at cluster edges, while data points in light gray are tilt angles of molecules inside the clusters. Black interval plots inside each dataset indicate mean value and 95% confidence interval.

We further investigated how FocA interactions within the clusters are influenced by lipid composition at the molecular scale. To increase the signal‐to‐noise ratio for the assignment of subunit interactions, we time‐averaged several consecutive frames in the HS‐AFM movies (**Figure** **3**a, left). In the resulting images, we first located the center of each FocA pentamer, as the geometrical average of the protomers, and determined the position of the five protomers relative to the center. Next, we identified nearest neighbor connections between adjacent FocA (Figure 3a, right). This allowed calculation of the interaction angles between protomers in FocA–FocA pairs using the center‐to‐center connection line as angular reference, and the position of the protomers as observable (Figure 3b). If the position of the closest protomer to the center along the connection line was in clockwise (CW) direction, we assigned a positive angle, if it was in counterclockwise (CCW) direction, we assigned a negative angle. Assuming a pentamer FocA formed a regular pentagon, the range of observable angles ranged from −36 to +36 degrees.

All determined intermolecular interaction angles were pooled in probability density maps and plotted for the various *E. coli* lipid environments (Figure 3c). We observed that with higher *E. coli* lipid proportions, the angle distribution tended to be more localized (Figure 3c, left, *χ* = 0.0), while in membranes with low *E. coli* lipid content, the angle distribution appeared more dispersed (Figure 3c, see *χ* > 0.0). This suggested that in high *E. coli* lipid environment, direct and specific protein–lipid–protein and/or protein–protein interactions are stabilized thereby restricting the movement of proteins within the cluster and leading to more confined protein–protein interactions at specific angles. Conversely, as the line tension of the protein clusters and protein–protein interaction strength decrease, proteins have more freedom to move and rotate within the cluster, resulting in a diverse pattern of interaction angles among protein pairs. Overall, FocA–FocA interactions predominantly favor orientations, where two protomers are aligned tip‐to‐tip, in line with the connection between the centers of the two proteins (Figure 3c, *χ* = 0.0, *). However, as *χ* increases to 0.5, proteins gain angular degree of freedom, allowing individual molecules to rotate relative to each other. This results in an angle distribution that comprises many interactions where the protomers are rotated with regard to the inter‐molecular connection and the tip‐to‐tip rule is only loosely followed (Figure 3c, *χ* = 0.50, **). As *χ* further increases, the molecules within the clusters acquire even more rotational freedom, leading to the occasional occurrence of tip‐to‐face configurations (Figure 3c, *χ* = 0.75, ***).

Next, to our surprise, we observed that some proteins within the clusters were tilted, i.e., as judged by comparing the height of individual protomers within a given pentamer. To determine the protein tilt angle, we identified the plane defined by the topographic position of the five FocA protomers and calculated the angle between the perpendicular axis to this plane and the *z*‐axis (Figure 3d, right, Experimental Section). We found that FocA in 100% *E. coli* lipid was more tilted (Figure 3d, top) than those in 20% *E. coli* lipids (Figure 3d, bottom). Assessing statistically the tilt of FocA within clusters in different lipid environments and in different locations within the cluster (Figure 3e), we found that proteins located at the cluster edges were more tilted (Figure 3e, dark gray) than those in the cluster center (Figure 3e, light gray). Indeed, it was noticeable that essentially only molecules at the cluster boundaries were affected by tilting and not those in the cluster center. Furthermore, protein displayed tilt at cluster boundaries scaled with higher *E. coli* lipid proportion (Figure 3e). Together, these results suggest that in *E. coli* lipids inter‐protein interactions are more specific (Figure 3c) leading to nonplanarity of the supramolecular architecture of FocA clusters, i.e., convex when viewed from the extracellular face, likely due to a slightly conical protein shape (Figure 3d, see *Discussion*). In contrast, in DOPC, where tight, specific protein–protein interactions are not favored, clusters are looser and flatter. These findings agree with the global cluster dynamics analysis, where proteins in high *E. coli* lipid content membranes had high line tension and low mobility, while clusters in high DOPC had low line tension and exhibited high mobility (Figure 2).

### 2.4 GlpF–GlpF Interactions are Lipid Dependent

**Figure 4:**
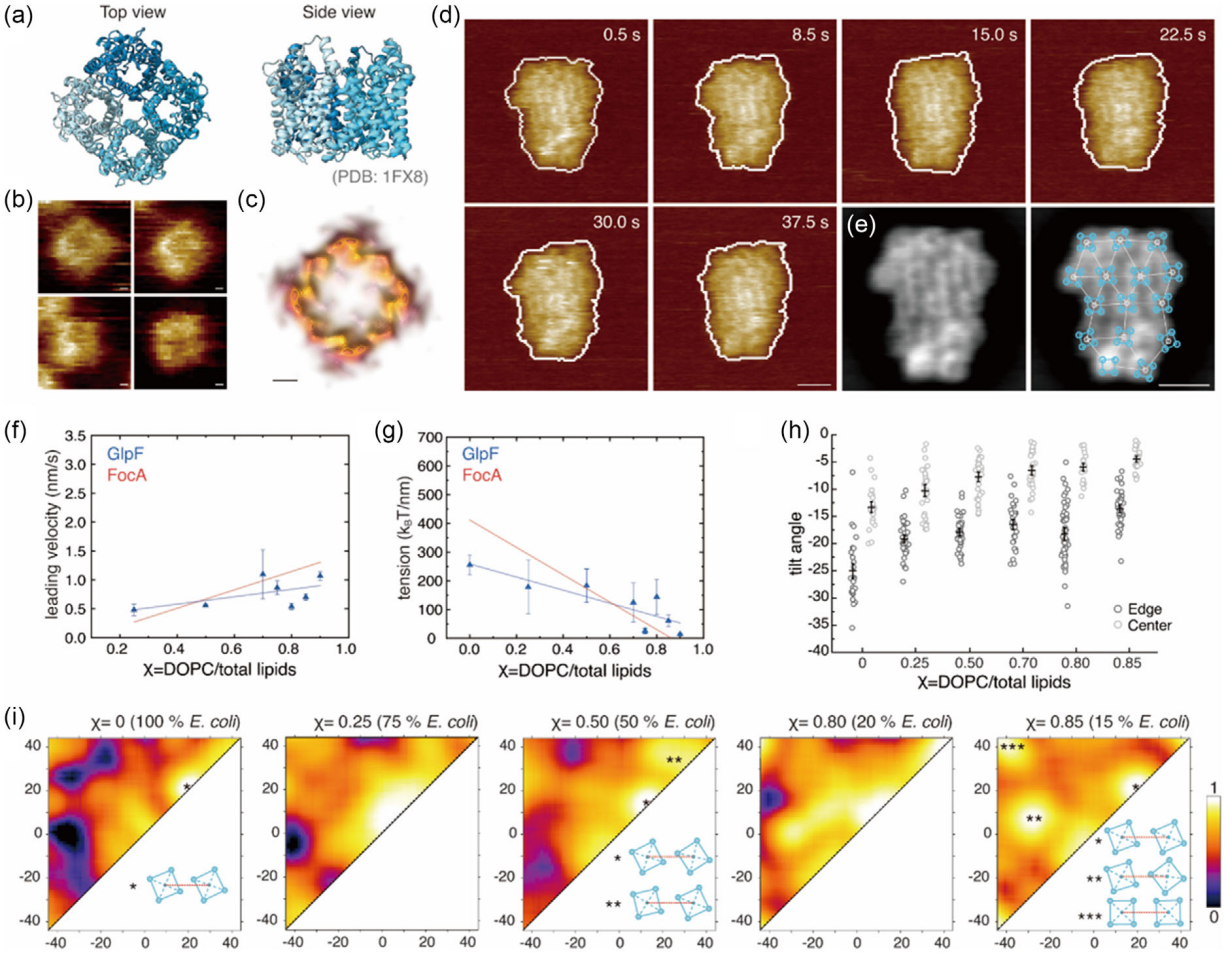
GlpF–GlpF molecular interactions are lipid modulated. a) Structure of GlpF tetramer in top (left) and side (right) view (PDB 1FX8). b) HS‐AFM images of individual GlpF tetramers. Scale bar: 1 nm. c) LAFM map of GlpF. Scale bar: 1 nm. d) HS‐AFM movie frames of GlpF cluster dynamics in 20% *E. coli* lipid environment (*χ* = 0.80). Scale bar: 10 nm. e) Time‐averaged frame of HS‐AFM movie in d) (left) with GlpF positions identification superimposed (right). White circles indicate the protein centers, white lines denote connecting lines between adjacent proteins, and blue circles represent the positions of the GlpF protomers. Scale bars: 15 nm. f) Mean leading‐edge velocity ($\mu_{v}$, nm s^−1^) and g) line tension, (*λ*, *k*_B_ *T* nm^−1^), as a function of DOPC/total lipids ratio *χ* ranging from 0 to 1 of GlpF clusters. Data points in (f,g) are from at least five protein clusters at each lipid condition (mean (marker), stdev (error bar)). Blue lines show trends of leading‐edge velocity (f) and line tension (g) as a function of lipid environment; red lines represent the trends for FocA clusters for comparison (see Figure 2h,i). h) Tilt analysis of proteins in GlpF clusters as a function of varying *χ* values of 0, 0.25, 0.50, 0.70, 0.80, and 0.85 (100, 75, 50, 30, 20, and 15% *E. coli* lipid, respectively). Tilt angles of molecules at the cluster edges in dark gray, while tilts of molecules in the cluster center are shown in light gray. The interval plot displays the mean value with 95% confidence interval. i) Probability density maps of GlpF–GlpF inter‐molecular interaction angles in different lipid environments in GlpF clusters. The range of angles analyzed within clusters containing tetrameric GlpF ranges from −45° to +45°. Representative GlpF–GlpF interaction angles are shown at the bottom right of panels *χ* = 0.0, 0.50, and 0.85.

Building on our investigations of the membrane‐dependent cluster dynamics and interactions of pentameric FocA, we wondered how a similar molecule with a different oligomeric state would behave. For this, we chose another *E. coli* membrane protein, the glycerol facilitator, GlpF. GlpF is essential for facilitating the diffusion of glycerol across the inner *E. coli* membrane, allowing bacteria to utilize glycerol as carbon and energy source for growth and survival.^[^ ^38^, ^39^ ^]^ Structural studies showed that the GlpF subunit adopts, like FocA, the aquaporin‐fold, but forms stable tetramers^[^ ^40^, ^41^ ^]^ (**Figure** **4**a). Thus, because both proteins have structural similarity being aquaporin‐like on the subunit level,^[^ ^42^, ^43^ ^]^ but adopt different oligomeric states, which we hypothesized should impact cluster and molecular interaction patterns and dynamics,^[^ ^44^, ^45^ ^]^ we studied tetrameric GlpF alone and together with FocA. HS‐AFM imaging confirmed the tetrameric, roughly square‐shaped assembly of GlpF (Figure 4b and Movie S4, Supporting Information**)**.^[^ ^46^ ^]^ Similar to FocA, GlpF molecules were uniformly inserted in the membrane in the HS‐AFM experiments. 3D‐LAFM resolved structural details of the helix‐connecting loops on the extracellular face (Figure 4c and S1b, Supporting Information) (Discussion). Thus, both FocA and GlpF reconstituted unidirectionally into the membrane with their extracellular faces exposed to the HS‐AFM tip, making them ideal proteins for our study (in contrast to AqpZ, which in our hands always reconstituted in up‐and‐down orientation).

HS‐AFM imaging of GlpF clusters revealed circumference time‐lapse dynamics similar to FocA clusters (Figure 4d **)**. Single‐molecule GlpF organization within the cluster was also revealed by the same analytical strategy (Figure 4e). We captured the dynamics of GlpF clusters in different lipid conditions and found, as observed in the FocA experiments, that the dynamics of the GlpF clusters decreased with increased *E. coli* lipid content (*χ* = 1.0–*χ* = 0.0) (Movie S5, Supporting Information). Identical to the behavior of FocA, GlpF clusters dissociated in 0% *E. coli* (*χ* = 0.0) lipids membrane (Figure S2b and Movie S6, Supporting Information). While FocA and GlpF shared similar qualitative trends, with decreasing fraction of *E. coli* lipid the mean leading‐edge velocity of protein clusters increased (Figure 4f) and line tension decreased (Figure 4g), quantitatively the changes were less pronounced in GlpF clusters than in FocA clusters. Indeed, GlpF clusters were more stable than FocA clusters in the same lipid conditions (Figure 4f). Also, the single‐molecule analysis showed similar interaction angle distribution trends for GlpF as for FocA in different lipid environments: The distribution of interaction angles between neighboring GlpF molecules widened as the fraction of *E. coli* lipid decreased (Figure 4i). However, the arrangement between GlpF molecules showed a different pattern compared to FocA–FocA interactions. At *χ* = 0.0, GlpFs preferred a face‐on arrangement, where the most probable interaction angles were distributed around (+20°, +20°), instead of (0°, 0°), corresponding to a tip‐to‐tip orientation as in FocA. In this arrangement, the sides of the tetramers, rather than their vertices, engage contact with the neighboring molecules (Figure 4i, *χ* = 0.0, *), approximating a regular tiling pattern. As the *χ* value increases, we observed a shift of the distribution toward more twisted face‐on arrangements (Figure 4i, *χ* = 0.50, **), and with further increase in *χ*, we observed tip‐to‐face configurations (Figure 4i, *χ* = 0.85, **), indicative of increased rotational freedom of the molecules at higher *χ*. Furthermore, we begin to observe substantial density representing complete face‐to‐face arrangements in the most mobile membrane characterized (Figure 4i, *χ* = 0.85, ***). Similar to FocA, GlpF at the cluster edges are more tilted than those in the center, and the overall tilt increases as the lipid environment has higher *E. coli* lipid content (Figure 4h). Moreover, the tilt of GlpF in GlpF clusters is significantly greater than FocA in FocA clusters in the same lipid conditions (compare Figure 4h vs 3e), resulting in a pronounced nonplanarity of the cluster morphology. In a 100% *E. coli* lipid environment (*χ* = 0.0), GlpF molecules at the cluster edges have a tilt angle up to ≈35°, whereas FocA was tilted up to ≈20°, suggesting that GlpF induced stronger membrane deformation when forming clusters as compared to FocA.

### 2.5 FocA–GlpF Mixed Clusters

**Figure 5:**
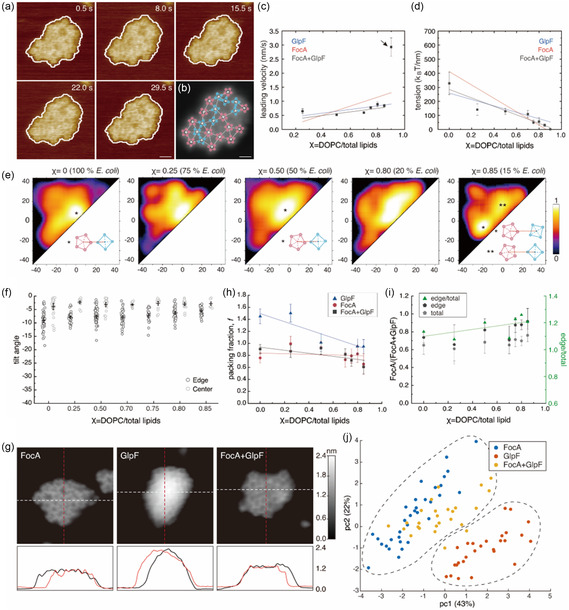
Mixed GlpF–FocA clusters. a) HS‐AFM movie frames of a mixted FocA–GlpF cluster in 20% *E. coli* lipid environment (*χ* = 0.80). Scale bar: 10 nm. The movie was captured at 2 frames per second. b) Time‐averaged frame of HS‐AFM movie in a) with GlpF and FocA positions identification superimposed. White circles indicate the protein centers, white lines denote connecting lines between adjacent proteins, and blue and pink circles represent the positions of GlpF and FocA protomers, respectively. Scale bars: 10 nm. c) FocA–GlpF cluster mean leading‐edge velocity ($\mu_{v}$, nm s^−1^) as a function of DOPC/total lipids ratio *χ* ranging from 0 to 1. Arrow indicates outlier data point in 10% *E. coli* lipid environment (*χ* = 0.90), indicative of FocA–GlpF cluster instability in low *E. coli* lipid content. d) FocA–GlpF cluster line tension, *λ*, *k*_B_ *T* nm^−1^ as a function of DOPC/total lipids ratio *χ* ranging from 0 to 1. Data points in c,d) are from at least five protein clusters at each lipid condition (mean (marker), stdev (error bar)). Gray lines in c,d) show increasing trend of leading‐edge velocity and decreasing trend of line tension as a function of *χ*. Red and blue lines represent trends of FocA and GlpF clusters, respectively, for comparison (see Figure 2h,i and 4f,g). e) Probability density maps of intermolecular interaction angles in different lipid environments of FocA–GlpF clusters. Representative interaction angles between GlpF–FocA are shown at the bottom right of panels *χ* = 0.0, 0.50, and 0.85. f) Tilt analysis of proteins in FocA–GlpF clusters at *χ* values of 0, 0.25, 0.50, 0.70, 0.75, 0.80, and 0.85 (100, 75, 50, 30, 25, 20, and 15% *E. coli* lipid). Tilt angles of molecules at the cluster edges in dark gray, while tilts of molecules in the cluster center are shown in light gray. The interval plot displays the mean value with 95% confidence interval. g) FocA–FocA (left), GlpF–GlpF (middle), and FocA–GlpF (right) cluster in 100% *E. coli* lipid environment (*χ* = 0.0). Bottom: Cross‐section profiles along horizontal (white) and vertical (red) lines illustrate cluster nonplanarity. h) Packing fraction, *f,* as a function of DOPC/total lipids ratio *χ* of FocA–FocA (red), GlpF–GlpF (blue), and mixed FocA–GlpF (gray) clusters. The lines show packing fraction trends, *f*, as function of *χ*. i) Moleculelar ratio of pentamer, FocA, in mixed FocA–GlpF clusters. Left *y*‐axis: FocA pentamer ratio at the cluster edge (dark gray) and in the total cluster area (light gray). Right *y*‐axis: Normalized FocA ratio at cluster edge versus total cluster area (green). Data points in (h,i) are from at least four protein clusters at each lipid condition (mean (marker), stdev (error bar)). j) Distribution of protein clusters (*n* = 103) in pc1–pc2 space of the principal component analysis (PCA). For each cluster, 8 distinct cluster features (Experimental Section) were measured and used as PCA input values. Dahsed lines: Two distinct catagories of protein clusters were found using a Gaussian Mixture Model. FocA–FocA (blue), GlpF–GlpF (red), and mixed FocA–GlpF (yellow) clusters. Clusters from all lipid environments were pooled for PCA.

Finally, we investigated mixed clusters of FocA and GlpF using the same methodology. When mixed, similar cluster dynamics at high % *E. coli* lipid condition (*χ* < 0.85) was observed (**Figure** **5**a, *χ* = 0.80), as compared to FocA‐only and GlpF‐only clusters. The time‐averaged HS‐AFM movie frame demonstrated the coexistence of pentameric FocA and tetrameric GlpF within the same clusters (Figure 5b). Observations of FocA and GlpF clusters in various lipid conditions also revealed membrane‐dependent differences in cluster mobility (Movie S7, Supporting Information). In agreement with our previous findings, increased *E coli* lipid content resulted in decreased mean leading‐edge velocity and increased line tension (Figure 5c,d). However, unlike the single‐component FocA or GlpF clusters, the distribution of interaction angles in the mixed FocA–GlpF experiments remained largely unaffected by changes in lipid membrane composition (Figure 5e). Notably, in environments with increasing *χ*, i.e., decreasing *E. coli* lipid fraction, the distribution did not broaden and showed no significant difference from the environment at *χ* = 0.0 (Figure 5e). This suggests that when molecules with different oligomeric states are mixed, the influence of the lipid environment is reduced.^[^ ^44^ ^]^ Also, the molecules in the mixed clusters had a much less pronounced tilt, <10° (Figure 5f), at the cluster edges in *χ* = 0.0, unlike the results from the single‐component systems with FocA (≈10°) or GlpF (≈25°) (Figure 5f,g). We hypothesize that the coexistence of molecules with different oligomeric states readily induces sufficient disorder within the clusters, rendering long‐range order—that would lead to nonplanarity of the clusters—impossible (Figure 5g).

To quantify the molecular packing, we measured the 2D packing fraction, *f*, as the area occupied by the molecules in the cluster over the total cluster area (Figure 5h).^[^ ^47^, ^48^ ^]^ All three types of clusters favored a denser packing at low *χ*, i.e., high *E coli* lipid content, indicative that the native lipids favored tight protein–protein interactions. For FocA‐only and FocA–GlpF clusters, the proteins covered ≈60–80% of the total cluster area at *χ* > 0.6, while the protein coverage surged to ≈80–100% at lower *χ*. GlpF‐only clusters, on the other hand, had an apparent 2D packing fraction *f* > 1 at low *χ* as a result of the pronounced nonplanarity of the clusters.

Notably, the mixed FocA–GlpF clusters contained a higher proportion of FocA than GlpF molecules (Figure 5i), as ≈60–70% of the total molecules were FocA in all lipid environments (Figure 5i, total). It is worth noting that FocA and GlpF were reconstituted at a 1:1 (w:w) ratio (Experimental Section), thus GlpF should in theory slightly out‐number FocA in the reconstituted proteo‐liposomes given its slightly smaller size. Therefore, FocA is favored in the the FocA–GlpF clusters. Besides, FocA and GlpF molecules were not uniformly distributed in the clusters, where FocA molecules favored the cluster edges by a factor of ≈1.1–1.2, in all lipid environments (Figure 5i, edge/total). These observations suggest that FocA molecules at the edges are crucial for stabilizing FocA–GlpF clusters: as membrane mobility increases (larger *χ*, more DOPC), a greater presence of FocA at the cluster edge is required for stable cluster formation, or, in other terms, GlpF likely dissociated from cluster edges under such conditions. To objectively categorize the protein clusters in all lipid environments, we analyzed 8 cluster morphology and dynamics characteristics in 103 clusters and performed a principal component analysis (PCA, Experimental Section). We found that the protein clusters distributed into two distinct groups in the pc1–pc2 space (Figure 5j), where one group comprised GlpF‐only clusters, while the other group primarily consisted of FocA‐only and most FocA–GlpF clusters. Thus, the FocA whose pentameric architecture prohibits regular tiling forms clusters that are morphologically and dynamically closer to mixed clusters, while the tiling‐compatible tetrameric GlpF forms substantially different clusters.

These results were further complemented by the analysis of center‐to‐center distances between molecules (**Table** **1**, *d*_GlpF‐GlpF_: GlpF‐only clusters, *d*_FocA‐FocA_: FocA‐only clusters, *d*_FocA‐GlpF_: FocA and GlpF mixed clusters). In the case of the mixed clusters, we categorized the distances into tetramer–tetramer, pentamer–pentamer, and pentamer–tetramer interactions. The distance measurements showed neglectable differences in the single‐component clusters, i.e., *d*_GlpF‐GlpF_ (average across all *χ*: 6.7 ± 0.2 nm) and *d*_FocA‐FocA_ (average across all *χ*: 7.8 ± 0.2 nm) as a function of *χ*. Also, the mean *d*_FocA‐FocA_ and *d*_GlpF‐GlpF_ center‐to‐center distances are similar in the mixed reconstitution as in the single‐component reconstitutions, with *d*_FocA‐GlpF_ (average across all *χ*: 7.2 ± 0.7 nm) lying between the homo‐molecular interactions, across all lipid environments (Table 1). However, molecules in the FocA–GlpF mixed clusters displayed enlarged center‐to‐center distances for all pairs *d*_FocA‐FocA_, *d*_GlpF‐GlpF_, and *d*_FocA‐GlpF_ at high *χ*. Especially, in the most mobile membranes (*χ* = 0.85, *E coli* % = 15%), where the center‐to‐center distances were ≈15% larger than in low *χ*. We hypothesize that in the mixed clusters, where many native lipids can be recruited in the packing spaces between pentameric FocA and tetrameric GlpF, clusters are particularly loose and at the brink of dissociation when *E coli* lipid content is low (Table 1).

**Table 1:** Center‐to‐center distances between molecules in GlpF clusters (*d*_GlpF‐GlpF_), in FocA clusters (*d*_FocA‐FocA_), and in mixed FocA/GlpF clusters (*d*_GlpF‐GlpF_, *d*_FocA‐FocA_, *d*_FocA‐GlpF_).

GlpF		FocA	
Lipid composition	*d*_GlpF‐GlpF_ (nm)	Lipid composition	*d*_FocA‐FocA_ (nm)
*χ* = 0.00 (100% *E. coli*)	6.6 ± 0.7 (*n* = 63)	*χ* = 0.00 (100% *E coli*)	7.8 ± 0.8 (*n* = 68)
*χ* = 0.25 (75% *E. coli*)	6.5 ± 0.6 (*n* = 121)	*χ* = 0.25 (75% *E coli*)	7.7 ± 0.7 (*n* = 113)
*χ* = 0.50 (50% *E. coli*)	6.8 ± 0.7 (*n* = 113)	*χ* = 0.50 (50% *E coli*)	7.6 ± 0.7 (*n* = 109)
*χ* = 0.80 (20% *E. coli*)	7.0 ± 0.6 (*n* = 116)	*χ* = 0.70 (30% *E coli*)	7.9 ± 0.6 (*n* = 95)
*χ* = 0.85 (15% *E. coli*)	6.7 ± 0.5 (*n* = 117)	*χ* = 0.85 (15% *E coli*)	8.1 ± 0.6 (*n* = 68)
Average	6.7 ± 0.2	Average	7.8 ± 0.2

FocA/GlpF			
Lipid composition	*d*_GlpF‐GlpF_ (nm)	*d*_FocA‐GlpF_ (nm)	*d*_FocA‐FocA_ (nm)
*χ* = 0.00 (100% *E. coli*)	6.1 ± 0.6 (*n* = 13)	6.9 ± 0.6 (*n* = 50)	7.1 ± 0.5 (*n* = 55)
*χ* = 0.25 (75% *E. coli*)	6.4 ± 1.0 (*n* = 8)	6.8 ± 0.7 (*n* = 39)	7.2 ± 0.7 (*n* = 29)
*χ* = 0.50 (50% *E. coli*)	6.3 ± 0.7 (*n* = 20)	6.8 ± 0.8 (*n* = 71)	7.2 ± 0.6 (*n* = 63)
*χ* = 0.70 (30% *E. coli*)	6.5 ± 0.8 (*n* = 14)	7.1 ± 0.9 (*n* = 35)	7.5 ± 0.6 (*n* = 23)
*χ* = 0.75 (25% *E. coli*)	6.8 ± 0.0 (*n* = 1)	7.4 ± 0.6 (*n* = 25)	8.1 ± 0.8 (*n* = 31)
*χ* = 0.80 (20% *E. coli*)	6.4 ± 0.8 (*n* = 10)	6.7 ± 0.6 (*n* = 46)	7.3 ± 0.7 (*n* = 33)
Low *χ* average	6.4 ± 0.2	6.9 ± 0.2	7.3 ± 0.3
*χ* = 0.85 (15% *E. coli*)	7.1 ± 1.5 (*n* = 3)	8.7 ± 1.7 (*n* = 17)	8.8 ± 1.3 (*n* = 19)
High *χ* average	7.1 ± 1.5	8.7 ± 1.7	8.8 ± 1.3
Two‐tailed *p* value (*)	0.006**	<0.0001****	<0.0001****
Average	6.5 ± 0.3	7.2 ± 0.7	7.6 ± 0.6

In mixed FocA–GlpF clusters, the center‐to‐center distances were categorized as GlpF–GlpF, FocA–FocA, and FocA–GlpF interactions. Values are the peak ± 1 standard deviation of Gaussian fits of the molecule center‐to‐center distance distributions, and *n* is the number of measured center‐to‐center distances, pooled from at least five protein clusters. The *p*‐values were calculated between the low *χ* population (*χ* ≤ 0.80, ≥20% *E. coli* lipid) and the high *χ* population. Asterisk indicates statistical significance: *p < 0.05, **p < 0.01, ***p < 0.001, ****p < 0.0001.

Indeed, the center‐to‐center distances are influenced by multiple factors. Logically, pentameric FocA has a larger radius than tetrameric GlpF, which leads to a larger average center‐to‐center distance (7.8 vs 6.7 nm) in both single‐component and mixed clusters (Table 1). Additionally, the spatial arrangement of the molecules affects the center‐to‐center distance, as tip‐to‐tip arrangements result in larger distances than tip‐to‐face and face‐to‐face arrangements. Mixing different oligomers further complicates the situation. Thus, interpreting the center‐to‐center distance solely based on a single factor is challenging. However, one key finding from this analysis is that when the percentage of *E. coli* lipids falls below a critical threshold, mixed FocA–GlpF clusters are more affected by the lipid environment than the single‐component clusters.

## 3 Discussion

While, with the recent progresses in cryogenic electron microscopy (cryo‐EM) solving membrane proteins structures has become quite common, an interesting and poorly explored field is membrane protein supramolecular architecture and its underlying guiding principles at the mesoscale. What governs the interactions between membrane proteins and how do these interactions depend on the lipid environment?

Here, we built a well‐controlled bottom‐up system, reconstituting the formate transporter FocA and the glycerol facilitator GlpF in supported lipid bilayers of various compositions, and imaged them using HS‐AFM. We captured the organization and real‐time dynamics of the interactions of these proteins, offering insights into the rules and mechanisms of membrane protein interactions. While the influence of the tip‐sample interaction in AFM experiments can never be entirely excluded, we performed HS‐AFM imaging using minimal force conditions, i.e., cantilever oscillation amplitude damping of ≈15%, optimized for the study of membrane‐mediated protein interactions.^[^ ^19^ ^]^ These studies revealed unperturbed free diffusion and interaction of another *E.coli* aquaporin‐fold protein, AqpZ. In our experiments, membrane spreading was allowed to reach full coverage of the scan area, and imaging was initiated only after an equilibration of the sample and HS‐AFM. This approach ensured that the observed clustering behavior and diffusion dynamics represent a quasi‐equilibrium state. Therefore, the protein behavior reported here reflects intrinsic lipid environment‐dependent properties.

Interestingly, in all HS‐AFM imaging experiments, we consistently observed both FocA and GlpF in a single orientation, namely with their extracellular domains facing the imaging buffer. This unidirectional insertion likely arises during the detergent‐removal mediated reconstitution process, where the presence of prominent hydrophilic domains on the extracellular side (Figure S4, Supporting Information) can promote preferential orientation.^[^ ^49^, ^50^ ^]^ As proteins reconstitute into the membrane, early membrane‐inserted channels may bias the orientation of subsequently reconstituted proteins. Additionally, the negatively charged mica may influence vesicle rupture and membrane spreading, or the preferential adsorption of one protein face. Together, these factors may explain the observed unidirectional insertion and support the reproducibility of our structural and dynamic analyses consistently analyzed on proteins exposing their extracellular face to the HS‐AFM tip.

Our results reveal that the lipid environment substantially influences membrane protein mobility and interactions within protein clusters. Increasing the *E. coli* lipid content results in increased stability of protein clusters, while environments enriched in DOPC lipids promote greater protein movement. To further understand our observation, the boundary fluctuation analysis provided quantitative measures of the cluster energetics. The line tension results indicate that protein clusters in lipid bilayers with higher *E. coli* content are more stable. Also, the supramolecular arrangement of FocA and GlpF within the clusters is sensitive to lipid composition, which is certainly the molecular basis of cluster stability and thus the measured line tension. High *E. coli* lipid content led to tight protein interactions, whereas scarceness of *E. coli* lipid led to greater assembly variability and looser packing. This shows that membrane protein–protein interactions are lipid modulated, affecting their structural organization and potentially their function.^[^ ^51^, ^52^ ^]^

While both proteins, FocA and GlpF, showed similar trends in response to lipid variations, we found quantitative differences. In brief, the tetrameric GlpF packed overall tighter in more convex clusters^[^ ^53^, ^54^ ^]^ with higher line tension than the pentameric FocA, highlighting the importance of considering the oligomeric state and the possibility of 2D‐tiling when studying membrane protein interactions,^[^ ^45^ ^]^ where the tetrameric GlpF assembles more tightly than the pentameric FocA that cannot 2D‐tile. The mixed FocA/GlpF clusters were further destabilized. At *χ* = 0.90 (10% *E. coli* lipid content), the mixed clusters had an outlier leading‐edge velocity data point, relating to low cluster line tension and indicative that they were close to dissociation.

While the boundary fluctuation analysis provides quantitative estimates of cluster energetics through line tension measurements, we recognize that these values may be influenced by membrane–substrate interactions, though the molecules were found to be able to diffuse in the membrane and thus we expect that a ≈5 Å thick buffer layer interspaces between mica and membrane.^[^ ^19^ ^]^ Nonetheless, the comparative trends we observe across different lipid compositions are robust and reproducible. Notably, these trends are consistent with molecular dynamics simulations^[^ ^33^, ^48^, ^52^, ^54^ ^]^ and the biophysical characteristics of the lipid components involved.^[^ ^2^, ^4^, ^5^, ^8^ ^]^ Thus, while the absolute values of line tension should be interpreted with caution, the overall conclusions regarding lipid‐mediated modulation of membrane protein clustering are meaningful.

In this study, we utilized the height information provided by the HS‐AFM topography to quantify not only lateral arrangements of protein protomers but also nonplanarity across protein clusters. Specifically, we defined a planar fit across the subunits within each oligomer using their measured heights, from which we calculated the degree of tilt. This analysis revealed that tilting was more pronounced at the periphery of clusters and under high *E. coli* lipid conditions. These localized tilts indicate that supramolecular protein assemblies can give rise to nonplanar features, likely reflecting local membrane curvature. While this approach does not reconstruct a full 3D volume of membrane‐embedded proteins, it provides a high‐resolution surface‐level view, integrating both in‐plane (lateral) and out‐of‐plane (vertical) structural dynamics. Our combined use of 2D protomer mapping and topographic tilt measurements enables a comprehensive assessment of spatial heterogeneity in clustering behavior, offering insights into how lipid composition modulates supramolecular organization and curvature within the membrane.

**Figure 6:**
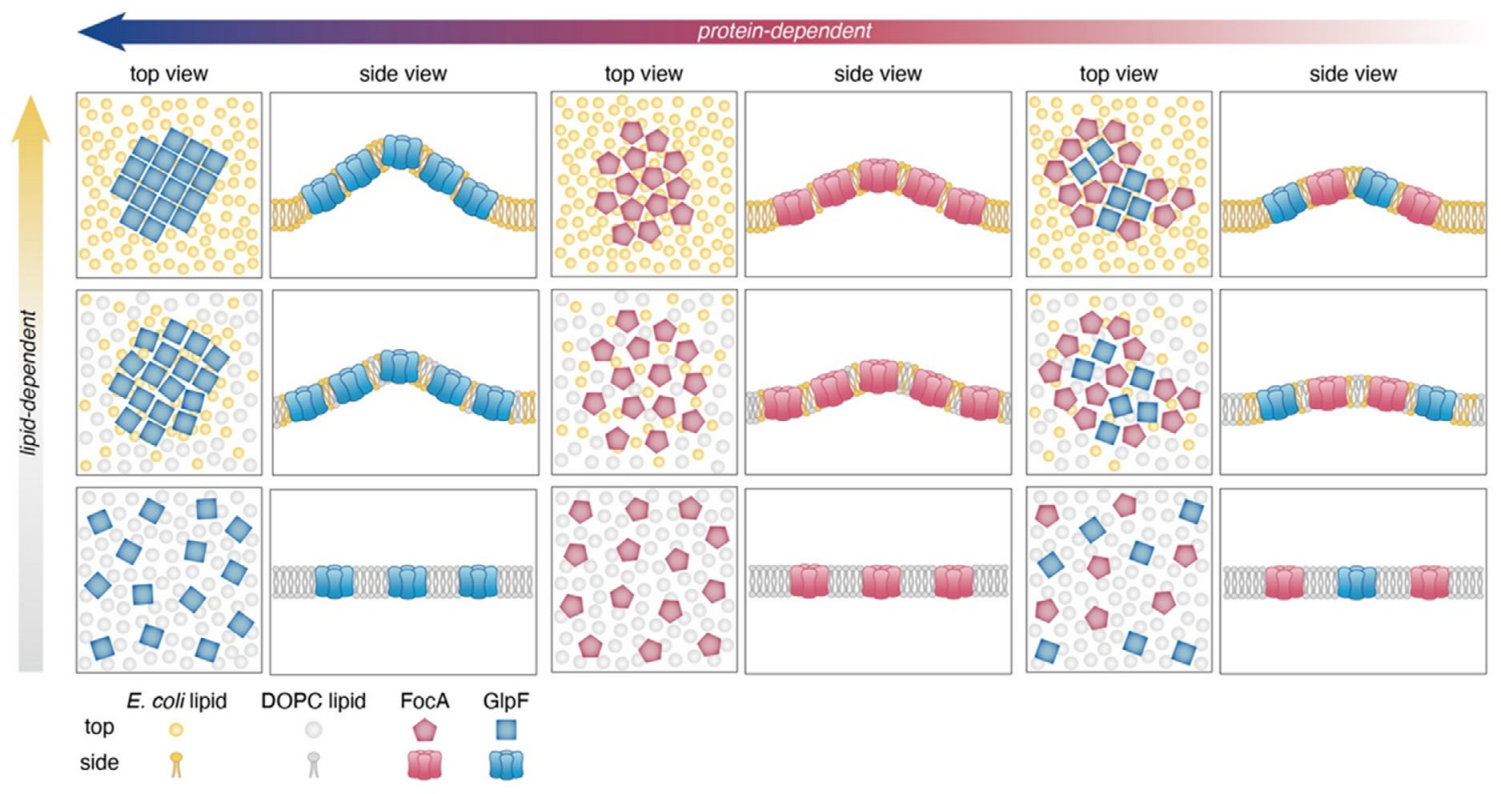
Lipid dependence and protein dependence of *E*_inclusion_ in membrane protein clustering. Protein clustering (*E*_clustering_) within membranes is determined by the solvent and inclusion energy density, *E*_solvent_ and *E*_inclusion_, respectively. *E*_inclusion_ is influenced by both, the lipid composition and the inherent properties of the proteins themselves. Lipid‐dependent effects include protein–lipid interactions and lipid‐mediated protein interactions, while protein‐dependent effects include structure and oligomeric state (ability to tile). Thus, protein clustering is a result of both membrane and protein characteristics, giving a large range of parameters such as protein expression and lipid metabolism that may govern cellular membrane organization.

Summarizing, we propose the following membrane protein clustering model based on our data (**Figure** **6**): The energy involved in protein clustering, *E*_clustering_, as evaluated by protein cluster line tension, *λ*, measurements in our experiments, is a result of the difference in energy density (*E*, in *k*_B_T nm^−2^) between the solvent, *E*_solvent_, and the inclusion, *E*_inclusions_, as: *λ* ∝ (*E*_solvent_ – *E*_inclusions_).

*E*_solvent_ characterizes energy related to the lipid environment, mainly lipid‐lipid interactions in an empty membrane, while *E*_inclusions_ represents the energies raised from interactions organizing lipid molecules and proteins within the protein cluster. The balance between these two interactions maintains protein clustering. When the two energy densities are equal, and the line tension decreases to zero, the inclusion dissolves. To test the energy densities of the membrane in the different lipid conditions, we performed molecular dynamics simulations (Data S1, Supporting Information). We found that while *E. coli* lipid formed a denser structure due to the presence of phosphatidylethanolamine (PE) with a smaller headgroup as compared to DOPC, the resulting energy density was very similar, likely due to favorable interactions between the zwitterionic headgroups: *E*_solvent*–*Ecoli_ ≈ *E*_solvent*‐*DOPC_.

Therefore, it is important to evaluate *E*_inclusions_, which arises from protein–lipid interactions, lipid‐mediated protein interactions, as well as direct protein–protein interactions: When *E. coli* lipids are present in sufficient quantities (>20%, *χ* < 0.80), the protein‐membrane system can recruit specific lipids to the protein interfaces to minimize energy costs (<*E*_inclusions_). For instance, specific protein interfaces may prefer shorter or longer lipids, cone‐shaped lipids, or lipids with specific charge (Figure S4, Supporting Information). *E. coli* lipids can offer such preferences as they are a mixture of phosphatidylglycerol (PG), PE, and cardiolipin (CL).^[^ ^55^ ^]^ These lipids may promote particular modes of protein association by lowering interfacial energy at defined contact sites, leading to specific protein–protein interactions. In contrast, DOPC is a single‐component lipid with a zwitterionic headgroup and two unsaturated alkyl chains, lacking the chemical diversity required to promote such specific interactions. The availability of a diverse range of lipid molecules in *E. coli* lipids may thus promote protein–protein interactions, stabilizing clusters. In contrast, in low availability of *E. coli* lipids (<10%, *χ* > 0.90%) or pure DOPC, the availability of specific lipids is lost, disfavoring protein–protein interactions (>*E*_inclusions_). Here, we define specific protein–protein interactions as associations observed between membrane proteins with consistent interfaces, such as the tip‐to‐tip interactions between FocA molecules in high *E. coli* lipid conditions. The emergence of these specific interactions appears to be lipid‐mediated, and may result from several physico‐chemical mechanisms, including 1) hydrophobic thickness matching, 2) membrane elastic properties, 3) electrostatic complementarity (e.g., anionic PG or CL interacting with cationic residues), 4) hydrogen bonding (e.g., involving the PE headgroup), and 5) shape matching (e.g., conical lipids, PE and CL, fitting specific membrane or protein curvature). While we cannot deconvolute these contributions individually, our data demonstrate that lipid composition alone can shift protein organization from non‐specific to specific clustering, suggesting a strong environmental regulation of protein assembly behavior.

All HS‐AFM imaging experiments in this study were conducted under well‐controlled, physiologically relevant buffer conditions consisting of 100 mM Tris–HCl (pH 7.6) and 150 mM KCl (Experimental Section). At pH 7.6, DOPC retains its zwitterionic character with a net neutral charge, while *E. coli*‐derived lipids such as PG and CL are expected to carry net negative charges. Although the surface charge of proteins can vary with pH due to the protonation and deprotonation of amino acid side chains, the constant and neutral pH used in this study minimizes this variable. Therefore, the observed differences in protein dynamics and clustering behavior can be attributed to differences in lipid composition rather than pH fluctuations. The ionic strength provided by 150 mM KCl mimics intracellular conditions and ensures electrostatic screening. Under this condition, the Debye screening length is ≈0.8 nm, meaning that electrostatic interactions between charged lipid headgroups and protein surfaces are effectively shielded beyond this range (Experimental Section). According to classical Derjaguin–Landau–Verwey–Overbeek theory,^[^ ^56^, ^57^ ^]^ such ionic conditions suppress long‐range Coulombic interactions, allowing short‐range forces—such as hydrophobic, steric, and membrane‐mediated interactions—to dominate. Therefore, the observed lipid‐dependent protein clustering and dynamic organization likely arise not from long‐range electrostatics but from local interactions modulated by lipid composition. This interpretation supports the conclusion that changes in lipid chemistry, rather than ionic strength, primarily govern the supramolecular behaviors observed under our experimental conditions and in cells. Nonetheless, future studies exploring a broader range of pH and ionic strengths may provide further insight into the electrostatic regulation of protein–lipid and protein–protein interactions in mixed lipid membranes.

Our data further suggest that the oligomeric state of the protein plays also an important role in *E*_inclusions_. Tetrameric GlpF can readily form regular and periodic tiling (<*E*_inclusions‐GlpF_), whereas pentameric FocA lacks this geometric advantage (>*E*_inclusions‐FocA_) and must form 2D‐clusters with larger lipid‐filled intermolecular spaces. As a consequence, FocA clusters are more sensitive to lipid‐related effects.

As different oligomers are mixed, specific protein–protein contacts are further reduced (»*E*_inclusions‐mix_), manifested by diverse intermolecular distances, and enriched tessellation variety, also leaving more lipid‐filled spaces between molecules. Likely therefore mixed FocA–GlpF clusters become critically unstable at *χ* = 0.90. Nevertheless, we did not observe demixing of GlpF and FocA into separate clusters, suggesting similar protein–protein interaction potentials between the different pairs (GlpF–GlpF, FocA–FocA, and GlpF–FocA), and the overall protein–protein interactions are well balanced.^[^ ^58^ ^]^ Indeed, the packing fraction in FocA‐only and FocA–GlpF clusters is similar. Interestingly, the observed stabilizing role of FocA at the edges of mixed clusters suggests that FocA's pentameric structure and lack of 2D tiling symmetry likely contribute to its greater stability at cluster edges compared to GlpF's regular tetrameric symmetry, which is less adaptable to irregular boundary geometries. Furthermore, the *E. coli* lipid mixture, including PE, PG, and CL, plays a crucial role in stabilizing protein–protein interfaces. These lipids may provide electrostatic complementarity and/or conical geometry at cluster junctions. Together, these structural and lipid‐mediated factors influence the stability and organization of protein clusters, highlighting the importance of lipid composition in modulating protein interactions.

The observation of pronounced protein tilt at cluster edges, leading to a dome‐like convex architecture of the cluster, raises intriguing questions about the potential relevance of such structures in native biological membranes. While our SLB system is simplified, the nonplanar features may emerge in vivo in regions, such as at endocytic sites or microdomains enriched in specific lipids or proteins.^[^ ^59^, ^60^ ^]^ These curved environments could lead to the further recruitment or stabilization of curvature‐sensitive transmembrane proteins, or conversely, the clustering of curved membrane proteins might induce local membrane curvature. We propose that the tilt‐induced convexity of the membrane protein clusters likely modulates local membrane properties and protein dynamics, which could influence accessibility, interaction specificity, or diffusion behavior within these clusters. These observations underscore the potential physiological implications of membrane protein organization and provide a foundation for further investigations into how such structural features influence cellular processes in vivo.

The methodologies and findings presented here pave the way for further investigations into the roles of lipids, protein structure, and oligomerization in modulating membrane protein supramolecular organization. Future studies could explore a wider range of lipid compositions, which should be of particular interest for eukaryotic membrane proteins. Cholesterol and sphingolipids are known to strongly influence membrane properties and add another layer of complexity. As solving membrane protein structures becomes more routine, our focus naturally turns toward the mesoscale, the length scale of the many membrane proteins, where supercomplexes, supramolecular structures, and transient functional ensembles are relevant. Understanding the underlying mechanisms of these phenomena will contribute to a more complete picture of membrane protein structure and function in health and disease.

## 4 Experimental Section

4.1

4.1.1

##### Protein Expression

The pET21b‐6His‐tobacco etch virus protease (TEV)‐FocA and pET21b‐6His‐TEV‐GlpF plasmids were transformed into *E. coli* competent cell strain BL21(DE3) for protein overexpression. The protein expression and purification were modified from a previous protocol.^[^ ^19^, ^61^ ^]^ In short, the overnight *E. coli* culture was diluted 100‐fold into fresh Luria broth (LB) broth and grown to an optical density at 600 nm (OD) between 1.5 and 1.7. Protein expression was induced by adding 1 mM isopropyl‐β‐D‐1‐thio‐galacto‐pyranoside (IPTG), and cells were then incubated at 37 °C for 3 h at 180 rpm. Cells were harvested by centrifugation at 5000 g for 15 min. The cell pellet was washed with phosphate‐buffered saline (PBS) and resuspended in 1/100 culture volume of a lysis buffer containing 50 mM Tris–HCl at pH 8.0, 100 mM NaCl, 1 mM ethylenediaminetetraacetic acid (EDTA), 1 mM phenylmethylsulfonyl fluoride (Merck), and 1 × Halt protease inhibitor cocktail (ThermoFisher Scientific). Cells were broken by sonication on ice, followed by centrifugation at 5000 g for 20 min, and then membrane fragments were collected by centrifugation at 140 000 g for 1 h at 4 °C. The membrane pellet was then solubilized overnight at 4 °C in a solubilization buffer containing 50 mM Tris–HCl at pH 8.0, 100 mM NaCl, and 5% *n*‐dodecyl‐β‐D‐maltopyranoside (DDM). The insoluble material was then removed by centrifugation at 210 000 g for 30 min at 4 °C.

##### Protein Purification

FocA and GlpF were purified from the detergent‐solubilized supernatant by nickel affinity chromatography using 3 mL HisPur Ni‐NTA resin (ThermoFisher Scientific) per 1 L cell. The column was equilibrated with 5 column volumes (CV) of a binding buffer containing 100 mM Tris–HCl at pH 8.0, 150 mM NaCl, 0.1% DDM, and 100 mM imidazole. Following protein loading, the nonspecifically bound material was removed by washing the column with 10 CV of the binding buffer. Elution was performed with a 5 CV gradient from 0 to 100% of an elution buffer containing 100 mM Tris–HCl at pH 8.0, 150 mM NaCl, 0.1% DDM, and 500 mM imidazole. Fractions containing FocA or GlpF were pooled and concentrated for size‐exclusion chromatography (SEC) using a Superose 6 Increase SEC column (Cytiva) attached to an ÄKTA system (Cytiva), with SEC buffer of 100 mM Tris–HCl at pH 8.0, 150 mM NaCl, and 0.1% DDM. Fractions containing pure FocA or GlpF proteins were pooled. The 6‐His‐tag was then removed by digestion with TEV protease. Protease was added to the purified protein at a ratio of 1:5 (w/w), and the buffer was adjusted to contain 100 mM Tris–HCl at pH 8.0, 150 mM NaCl, 0.5 mM EDTA, 1 mM dithiothreitol (DTT), 20% glycerol, and 0.1% DDM. Digestion was allowed to continue overnight at room temperature. The cleaved GlpF or FocA proteins were then separated from the TEV protease by nickel affinity using a 1 mL HisPur Ni‐NTA resin. The column was equilibrated with a wash buffer containing 100 mM Tris–HCl at pH 8.0, 150 mM NaCl, and 0.1% DDM. After sample loading, the column was washed with an additional 5 CV of the wash buffer. The proteins in the flow‐through were pooled and concentrated to a final concentration of 1 mg ml^−1^ with 20% of glycerol and stored at −80 °C.

##### Protein Reconstitution

Purified proteins were combined with a lipid mixture consisting of DOPC, 1,2‐dioleoyl‐sn‐glycero‐3‐phosphoethanolamine (DOPE), and 1,2‐di‐(9Z‐octadecenoyl)‐sn‐glycero‐3‐phospho‐L‐serine (DOPS) at a ratio of 8:1:1 (w:w:w) in a buffer containing >3 critical micelle concentration (CMC) of DDM (100 mM Tris–HCl at pH 7.6, 150 mM KCl, and DDM). The LPR ratio for the single FocA and single GlpF reconstitution was kept at 0.2 and for the mixture FocA&GlpF reconstitution was 0.3, with a final protein concentration of 0.5 mg ml^−1^. This mixture was stabilized for 2–3 h at 4 °C and then dialyzed for 12 h using cassettes (10 kDa, ThermoFisher Scientific) at room temperature against 500 mL of buffer (100 mM Tris–HCl at pH 7.6, 150 mM KCl, and 20% glycerol) to remove the detergent.

##### Lipid Preparation

*E. coli* total lipid and DOPC lipid solubilized in chloroform were purchased from Avanti Polar Lipids. The lipid solution in chloroform was dried using nitrogen gas and further dried for an additional 12 h in a vacuum environment. The dried lipids were rehydrated with imaging buffer (100 mM Tris–HCl at pH 7.6 and 150 mM KCl). To form small unilamellar vesicles (SUVs), the rehydrated lipids were then tip‐sonicated for 2 min. For HS‐AFM membrane extension and lipid fusion experiments, the lipids were diluted to a final concentration of 1 mg ml^−1^. SUVs were then added to the liquid cell chamber to facilitate membrane fusion and extension.

##### High‐Speed Atomic Force Microscopy (HS‐AFM) Imaging

All movies were obtained using HS‐AFM (SS‐NEX, RIBM) operated in amplitude modulation mode^[^ ^62^ ^]^ with a typical free amplitude (*A*_free_) of 1.0 nm and a setpoint amplitude (*A*_set_) of 0.9 nm. Ultrashort cantilevers (USC‐F1.5‐K0.6, Nanoworld) with a nominal spring constant of 0.60 N m^−1^ and a resonance frequency of ≈550 kHz in liquid were used. The mica surface on which the sample was physisorbed was immersed in a liquid chamber filled with imaging buffer (100 mM Tris–HCl at pH 7.6, 150 mM KCl) to perform HS‐AFM imaging under liquid conditions. All imaging experiments were carried out at laboratory temperature (25 °C).

For protein physisorption on a surface, 2 μl of the sample diluted with physisorption buffer (100 mM Tris–HCl at pH 7.6, 150 mM KCl, and 20 mM MgCl_2_) was applied onto freshly cleaved mica and incubated for 10 min for physisorption. After the incubation period, any excess nonphysisorbed sample was washed away with the imaging buffer (100 mM Tris–HCl at pH 7.6, 150 mM KCl).

For recording the lipid fusion process on the proteoliposomes, imaging was performed at a speed of 1 s f^−1^ with a scan area between 150 × 150 nm and 200 × 200 nm and a resolution of 200 × 200 pixels–300 × 300 pixels. To record the movement of single‐protein clusters, the scan size was reduced to 60 × 60 nm–80 × 80 nm, and the pixel resolution was adjusted to 120 × 120 pixels–160 × 160 pixels, with imaging at a speed of 0.5 s f^−1^.

HS‐AFM movies were aligned, flattened, and calibrated using home written ImageJ plugins (ImageJ, NIH).

##### Preparation of Poly‐L‐Lysine Coated Surface for Single Protein Imaging

Mica surface coated with Poly‐L‐Lysine (PLL) was prepared as previously described following the membrane‐extension membrane protein reconstitution (MEMPR) strategy^[^ ^29^ ^]^: 2 μL of PLL diluted to 0.001% was incubated on the mica surface for 2 min to ensure adsorption. The low concentration of PLL was employed to prevent the formation of multi‐PLL layers. Subsequently, the excess PLL was removed by washing with MQ water, and the PLL‐coated mica was allowed to dry in the air.

For protein sample intended for single protein imaging, reconstituted protein (0.5 mg ml^−1^) was incubated with DOPC lipid (0.5 mg ml^−1^) in a physisorption buffer containing 100 mM Tris–HCl at pH 7.6, 150 mM KCl, 20 mM MgCl_2_, and 1 × CMC DDM for 10 min. The sample solution after incubation was then applied to the PLL‐coated mica for an additional 10 min to ensure the fixation of the proteins followed by washing extensively with water to remove the detergent.

##### HS‐AFM Protein Cluster Dynamics Analysis

Codes for HS‐AFM cluster dynamics analysis were developed using home‐written MATLAB scripts. In short, the mask corresponding to the membrane protein cluster in each HS‐AFM frame was roughly calculated using the ImageJ built‐in ‘isodata’ threshold algorithm to generate a cluster ‐mask stack, which was then fine tuned in MATLAB. In this process, a three‐frame walking average strategy was applied to the rough cluster‐mask stack to remove pixels due to imaging noise. In parallel, the masks in all frames were aligned once more with respect to the cluster‐mask center. The final mask should capture the major morphology of the protein cluster and contain no “gap pixels” within it. Then, the contours of the cluster masks were calculated to generate the time‐resolved radial profile of the cluster, R(θ,t), recording the most probable radius *R* of the cluster at angle *θ* and time *t*. From R(θ,t), the mean leading velocity $\mu_{v}$ was calculated as

(2) $$\mu_{v}=\frac{\delta t\delta \theta }{2\pi T}\sum_{\theta =0}^{2\pi /\delta \theta }\sum_{t=0}^{T/\delta t}\left|R\left(\theta ,t+\delta t\right)-R\left(\theta ,t\right)\right|$$

The cluster line tension calculation was determined by Fourier analysis of capillary waves as showcased in the line tension calculation in lipid monolayers.^[^ ^35^ ^]^ In our analysis, we modeled the morphological fluctuation of the cluster when the system was at equilibrium as capillary waves and hence determined the relative radial profile of the cluster R′(θ), as

(3) R′(θ)=(R(θ,t+δt)−R(θ,t))/R(θ,t)

The relative radial profile of the cluster R′(θ) was fitted with the Fourier series expansion formula, as.

(4) $$R\prime \left(\theta \right)=1+a_{0}+\sum_{k=1}^{\infty }[a_{k}\;\cos\left(k\theta \right)]+\sum_{k=1}^{\infty }[b_{k}\;\sin\left(k\theta \right)]$$

where *k* is the mode number and $a_{k}$ and $b_{k}$ are the Fourier coefficients.^[^ ^35^ ^]^ At last, the relation between the mode number *k* (for *k* ≥ 2), the Fourier coefficients $a_{k}$ and $b_{k}$, the average cluster or domain radius $r_{0}$, and the line tension *λ* is.
(5)

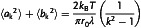




Therefore, the line tension *λ* could be extracted by the linear regression to this relation.

##### HS‐AFM Protein Tilt and Nearest‐Neighbor Analysis

Codes for HS‐AFM protein tilt and nearest‐neighbor analysis were developed using home‐written MATLAB scripts. The individual proteins of interest (particles) in the cluster were hand‐picked from flattened HS‐AFM frames, from which the rough coordinates of proteins were exported to MATLAB. To determine the exact protein positions in the cluster as well as determine the protomer positions, images of individual particles were cropped from the HS‐AFM frames according to their rough coordinates. The particle images were then rotationally symmetrized based on the protein molecular symmetry (*nf*, *nf* = 4 for tetrameric GlpF and *nf* = 5 for pentameric FocA) at various origins near the image center, and an internal symmetry value, isv, was calculated at each origin, where isv is the cross‐correlation value between the pre‐ and post‐symmetrized images. The origin of the highest isv was supplemented with the rough particle position in the cluster to give the final position of each particle. In this process, the positions of individual protomers of a particle were also extracted.

To determine the tilt *ω* of each particle, we first determined the most likely plane formed by the protomers, given the protomer positions *x*, *y* and height measurements *h*, from which the normal vector was defined. The tilt is then calculated as the acute angle between the normal vector and the *z*‐axis.

For nearest‐neighbor analysis, we first used Delaunay triangulation to identify neighbor particle pairs based on the final particle positions within the cluster. These particle pairs were then manually checked in the HS‐AFM images to confirm the presence of physical protein–protein interactions, and then incorrect pairs were eliminated. For each confirmed particle pair, we measured the center‐to‐center distance, *d*, and identified the interacting protomers. The interacting protomer in each paired particle is the one with the smallest angular separation to the center‐to‐center connection of the pair. Finally, we analyzed the interaction angles, *α* and *β*, defined as the angles between the interacting protomer of one paired particle and the center position of the other particle.

##### HS‐AFM Protein Cluster 2D Packing Fraction Analysis

The packing fraction, *f*, of the protein clusters was estimated from 1). Cluster area derived from the cluster‐mask (*A*_cluster_), 2). Number of single molecules (*n*), and 3). single‐molecule cross‐section areas. The single‐molecule areas were derived using two independent strategies. We first derived the single‐molecule cross‐section areas from the HS‐AFM imaging data of isolated, immobilized FocA and GlpF molecules. In parallel, we analyzed the 3D‐LAFM density map and PDB structures of the corresponding molecules. These two strategies rendered similar results in which GlpF has a cross‐section area (*A*_GlpF_) of ≈38 nm^−2^, and FocA has a cross‐section area (*A*_FocA_) of ≈43 nm^−2^. The 2D packing fraction was calculated as.^[^ ^47^, ^48^ ^]^

(6) $$f=(n_{\text{GlpF}}A_{\text{GlpF}}+n_{\text{FocA}}A_{\text{FocA}})/A_{\text{cluster}}$$

##### Principal Component Analysis (PCA)

To categorize the protein clusters, a PCA workflow was developed using home‐written MATLAB scripts. From the cluster data, 8 features were extracted: 1) Total number of proteins in the cluster. 2) The ratio of GlpF/FocA molecules at the cluster edge. 3) The ratio of total GlpF/FocA molecules in the cluster (edge + center). 4) The ratio of DOPC/*E.coli* lipids the cluster was observed. 5) The 2D packing fraction. 6) The eccentricity of the cluster morphology derived from an ellipse best fit to the cluster circumference. 7) The line tension at the cluster edge. 8) The leading velocity at the cluster edge. These features from all analyzed clusters in all lipid environments (*n* = 103) were tabulated into a 103‐by‐8 data matrix for a standard PCA^[^ ^63^ ^]^ analysis performed with the MATLAB built‐in ‘*pca*’ function series. The variances of the first two principal components (pc1 and pc2), ordered in decreasing variance values, are 43 and 22% respectively, accounting for ≈65% of the total variance of the protein cluster dataset. In contrast, the third pc has a variance of ≈13%, thus neglectable for the PCA analysis. A Gaussian Mixture Model (GMM)^[^ ^64^ ^]^ clustering algorithm was applied to group protein clusters into two major groups.

##### Fluorescence Recovery after Photobleaching (FRAP) Experiment

For the FRAP experiment, *E. coli* total lipid, DOPC lipid, and NBD‐PC lipid dissolved in chloroform were mixed under the following conditions: 1) *E. coli* total: DOPC: NBD‐PC 98:0:2 (w:w:w), 2) 50:48:2 (w:w:w), 3) 0:98:2 (w:w:w). Each lipid mixture underwent nitrogen drying, vacuum drying, and rehydration with imaging buffer following the same procedure as before to obtain SUVs. The lipid suspension was bath‐sonicated for 30 min,^[^ ^37^ ^]^ and the final lipid concentration was adjusted to 1 mg ml^−1^. To minimize the photobleaching of NBD‐PC, samples were prepared immediately before the experiment.

The prepared lipid samples were deposited onto glass coverslips (No. 1.5) that had been cleaned for over an hour with sonication. 50 μl of the sample was incubated for 10 min to allow SUVs to deposit onto the coverslip. Subsequently, the coverslips were rinsed with imaging buffer, and the coverslips containing SUVs were supported by glass slides. During FRAP imaging, the glass slides were flipped over to position the coverslips closer to the objective lens.

To record the fluorescence of NBD‐PC, a laser with an excitation wavelength of 474 nm and an emission wavelength of 533 nm was employed. Photobleaching was executed for 50 s using a 405 nm laser at maximum power. The total imaging scan area before photobleaching was 32.4 μm × 32.4 μm, and photobleaching was performed by setting a region of interest (ROI) circle with a diameter of 10 μm. Imaging acquisition conditions involved capturing 1 frame per second before photobleaching, resulting in a total of 10 frames. After photobleaching, 1 frame was captured every 5 s for an additional 10 frames. Subsequently, to minimize photobleaching induced by the laser, 1 frame was captured every 30 s, amounting to a total of 13 frames. FRAP experiments were conducted in at least three different positions for each lipid condition. All FRAP experiments were performed at laboratory temperature (25 °C) to ensure consistency with HS‐AFM imaging conditions.

##### FRAP Data Analysis

Codes for FRAP analysis were developed using home‐written MATLAB scripts. In short, the post‐bleach fluorescence intensities were normalized to the pre‐bleach background value. The diffusion coefficient, *D*, was extracted following the standard protocol,^[^ ^65^ ^]^ as
(7)

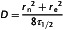

where $\tau_{1/2}$ is the half‐time of postbleach fluorescence recovery, $r_{n}$ is the normal radius in the FRAP experiments, and $r_{e}$ is the effective radius computed from the normalized mean postbleach profile f(x) across the bleach ROI, as 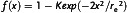.

##### Debye Length Calculation

Under the HS‐AFM imaging conditions (100 mM Tris–HCl (pH 7.6) and 150 mM KCl at 25 °C), the electrostatic screening of charges in the system can be characterized by the Debye length ($\lambda_{D}$), which quantifies the distance over which electrostatic interactions are screened in an electrolyte solution. The Debye length is calculated using the following equation.^[^ ^66^ ^]^

(8) $$\lambda_{D}=\sqrt{\frac{\varepsilon_{r}\varepsilon_{0}k_{B}T}{2N_{A}e^{2}I}}$$

where $\varepsilon_{r}$ is the relative permittivity (dielectric constant) of the medium (≈78.5 for water at 25 °C), $\varepsilon_{0}$ is the vacuum permittivity (8.854 × 10^−12^ F m^−1^), $k_{B}$ is the Boltzmann constant (1.381 × 10^−23^ J K^−1^), *T* is the absolute temperature in Kelvin (298 K for 25 °C), $N_{A}$ is Avogadro's number (6.022 × 10^−23^ mol^−1^), *e* is the elementary charge (1.602 × 10^−19^ C), and *I* is the ionic strength of the solution (in mol L^−1^).

For a monovalent electrolyte like KCl at a concentration of 150 mM, the ionic strength *I* is 0.15 mol L^−1^. Plugging these values into the above equation yields a Debye length of ≈0.8 nm.

## Code Availability

The codes for the protein cluster analysis are available on GitHub (https://github.com/rafaeljiang23/ProteinClusterAnalysis). The codes for all other analysis are available from the authors upon reasonable request.

## Conflict of Interest

The authors declare no conflict of interest.

## Author Contributions

**Eunji Shin, Yining Jiang, Batiste Thienpont, James N. Sturgis**, and **Simon Scheuring** designed the study. **Eunji Shin** and **Batiste Thienpont** purified the protein. **Eunji Shin** reconstituted the protein and performed the HS‐AFM experiments. **Yining Jiang** developed the protein cluster analysis workflow and codes. **Eunji Shin** and **Yining** **Jiang** analyzed the HS‐AFM data. **Eunji Shin**, **Yining Jiang**, and **Simon Scheuring** wrote the article. All authors edited the manuscript. and **Simon Scheuring** supervised the project.

## Supporting information

Supplementary Material

## Data Availability

The data that support the findings of this study are available from the corresponding author upon reasonable request.
