# Supplementary material for: High‐Speed Atomic Force Microscopy Reveals the Dynamic Interplay of Membrane Proteins is Lipid‐Modulated
Source: Small Sci. 2025 Jul 8;5(9):2500258. doi: 10.1002/smsc.202500258 (PMC12412613; doi:10.1002/smsc.202500258)
Supplement: Supplementary file 1 — Supplementary Material [file SMSC-5-2500258-s001.zip › Supplementary Material_2025_05_02_Small_Sci..pdf]

Supplementary Material

High-Speed AFM Reveals the Dynamic Interplay of Membrane Proteins is Lipid-Modulated

Authors

Eunji Shin[1,†], Yining Jiang[1,†], Batiste Thienpont[2], James N Sturgis[2], and Simon Scheuring[1,3*]

Affiliations

[1] Weill Cornell Medicine, Department of Anesthesiology, 1300 York Avenue, New York, NY 10065, USA.

[2] Laboratoire d'Ingénierie des Systèmes Macromoléculaires (LISM), Unité Mixte de Recherche (UMR) 7255, Centre National de la Recherche Scientifique (CNRS), Aix Marseille Université, Marseille, France.

[3] Weill Cornell Medicine, Department of Physiology and Biophysics, 1300 York Avenue, New York, NY 10065, USA.

† Equal author contribution

* Correspondence to: sis2019@med.cornell.edu

This PDF file includes:

Supplementary Figure 1 to 4
Supplementary Data 1
Supplementary Data References

Other Supplementary Materials for this manuscript include the following:

Supplementary Movie 1 to Movie 7

Supplementary Figure 1

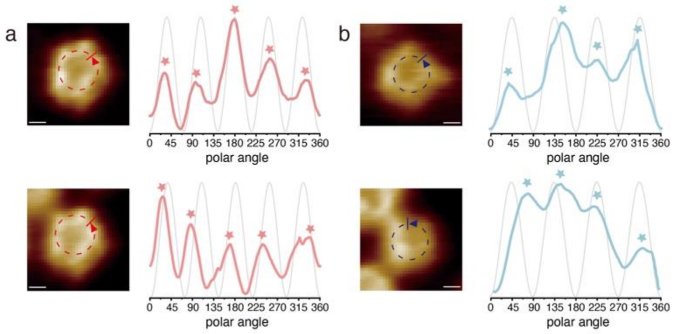

Supplementary Figure 1 | Protein profiling: FocA and GlpF at the single molecule level. Individual **(a)** FocA, and **(b)** GlpF molecules. Time-averaged HS-AFM movie frames (left, scale bars: 2 nm), and polar angle profile plots (right). Height profiles were plotted in counter-clockwise direction as indicated in the left image. The asterisks indicate profile peak positions: 5 major peaks were found for FocA with ~72º periodicity (sine fit) (a), and 4 major peaks for GlpF with ~90º periodicity (see sine fit) (b).

Supplementary Figure 2

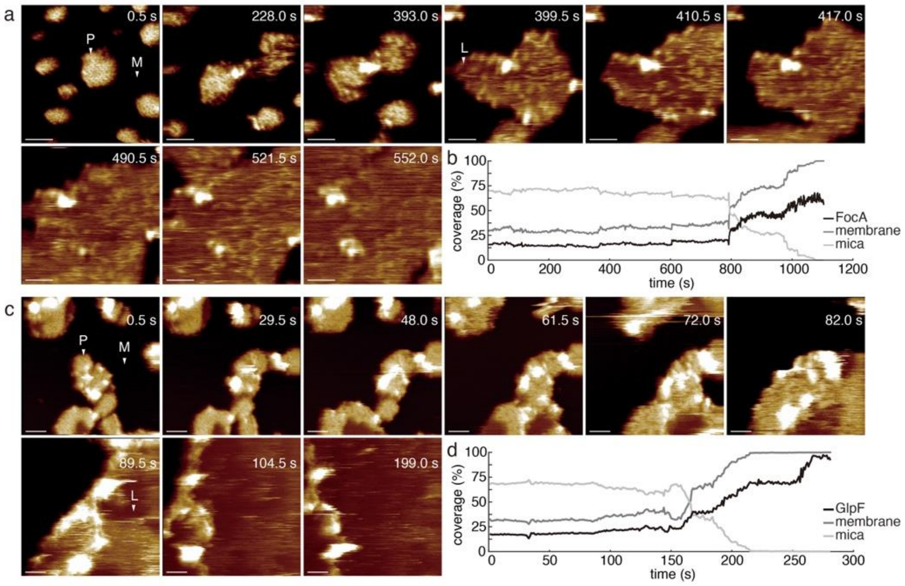

Supplementary Figure 2 | Both FocA and GlpF exhibited dissociation upon the introduction of 100% DOPC lipid. (a, c) HS-AFM movie frames visualizing the process of lipid membrane fusion of (a) reconstituted FocA and (b) reconstituted GlpF on mica with the addition of 100% DOPC lipids. 'P' indicated the region of reconstituted protein liposomes, 'M' indicated the mica region, and 'L' represented the lipid membrane region. A time scale was provided in the upper right corner of each frame of the movie, and a scale bar of 30 nm was included at the bottom left of each frame. **(b, d)** Analysis of the coverage percentage in the HS-AFM movies for both reconstituted (b) FocA and (d) GlpF. The graph displays the coverage of the area occupied by membrane protein (either FocA or GlpF) with a black line, the area occupied by the lipid membrane with a dark gray line, and the area occupied by mica with a light gray line. This analysis allowed for the quantitative assessment of the behavior of FocA and GlpF in the presence of 100% DOPC lipid. Note, in the DOPC-only SLBs the membrane protein coverage appeared to increase (b, d, black lines) following bilayer fusion, while the protein content should remain roughly constant. This is due to the fact that HS-AFM is too slow to depict individual freely diffusing molecules and thus records them with a diffuse enlarged topography.

Supplementary Figure 3

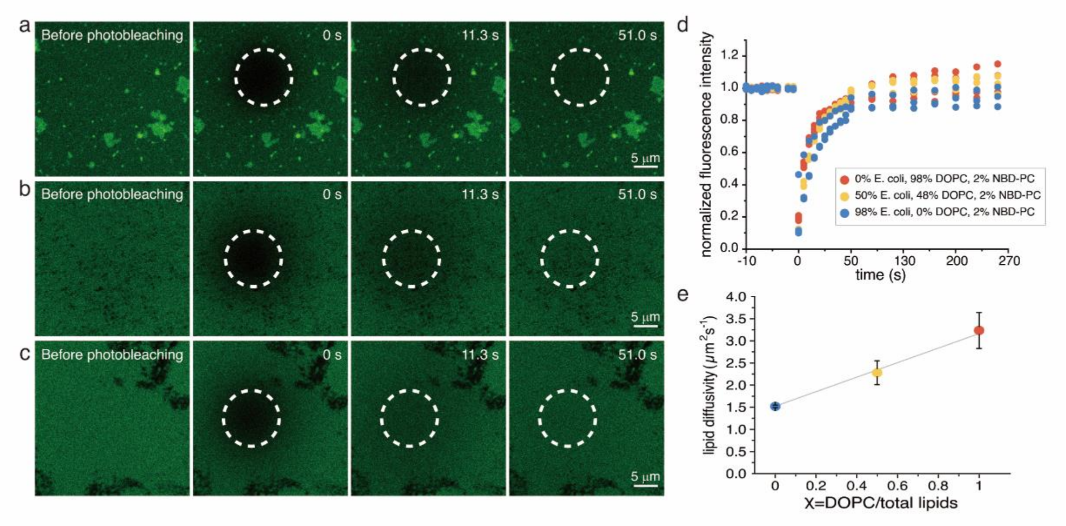

Supplementary Figure 3 | Fluorescence Recovery After Photobleaching (FRAP) Analysis of Lipid Membrane Dynamics Under Varied *E. coli* and DOPC Conditions. (a, c) Fluorescence recovery after photobleaching (FRAP) experiments on fluorescence-labeled membranes under the lipid conditions of *E. coli*: DOPC: NBD-PC **a** 98: 0: 2, **b** 50: 48: 2, **c** 0: 98: 2. The white circles indicate the photobleaching area. Fluorescence image frames at the same timeline were selected for each condition for comparison. **(d)** Time-lapse fluorescence intensity within the photobleaching area. Fluorescence intensity was normalized to the pre-bleaching background. To ensure statistical reliability, photobleaching experiments were conducted at three or more different spots for each lipid condition. **(e)** Diffusion coefficient plots for the three lipid conditions. Each data point represents the mean value obtained from three or more different FRAP areas, and error bars indicate the standard deviation. Data points colored blue, yellow, and red correspond to lipid conditions with *E. coli*: DOPC: NBD-PC 98: 0: 2, 50: 48: 2, and 0: 98: 2, respectively. Line shows trend of increasing lipid diffusivity with increases DOPC lipid portion. The data is the same as that in **Figure 2j**.

Supplementary Figure 4

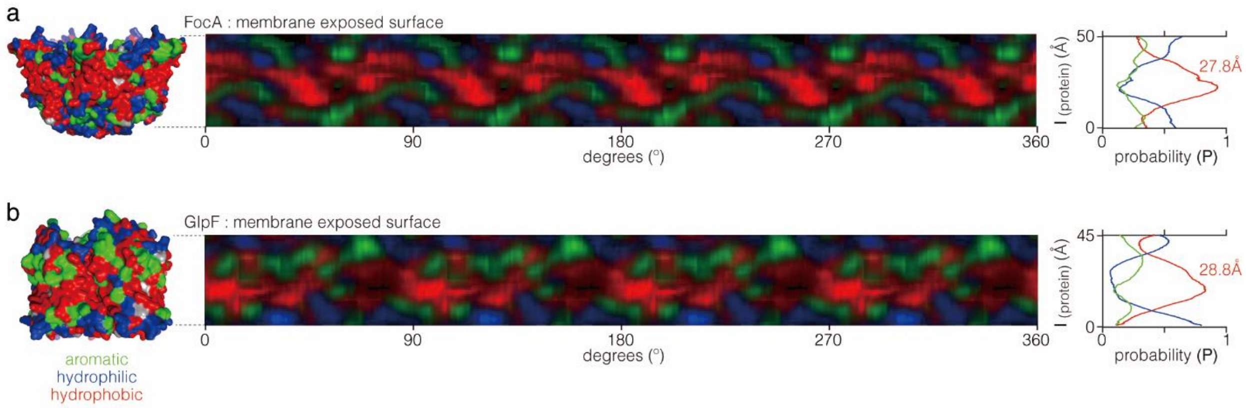

Supplementary Figure 4 | Hydrophobic thickness analysis of FocA and GlpF. (a) Membrane exposed surface of FocA (PDB 3KCV). **(b)** Membrane exposed surface of GlpF (PDB 1FX8). Left: Surface representation of the structure. Middle: 'unrolled' surface as a function of polar coordinate. Right: plot of the relative abundance of hydrophilic, hydrophobic and aromatic surface exposed residue surfaces as a function of the protein thickness z-axis. Hydrophobic thickness analysis was performed as in Jiang *et al*. 2022 [(23)]. The hydrophobic thickness, FocA: 27.8 Å and GlpF: 28.8 Å, was determined as $A_{hydrophobic}/c_{surface}$, where $A_{hydrophobic}$ represents the area of the hydrophobic pixels on the 'unrolled' surface and $c_{surface}$ is the width of the 'unrolled' surface. Residue coloring in all panels: hydrophilic (blue), hydrophobic (red), and aromatic (green).

Supplementary Data 1

To estimate $E_{solvent}$ under varying lipid conditions DOPC/(*E. coli* lipids + DOPC), χ, we performed molecular dynamics simulation and assessed the packing of the lipid molecules. To simulate the *E. coli* lipid membrane, we generated a bilayer with composition: POPE:POPG:CL 53:27:20 (w/w), equivalent to POPE:POPG:CL 59:30:11 mol/mol. Cardiolipin was modelled with SN1 chains palmitoyl and SN2 chains oleoyl. This corresponds approximately to the lipid composition measured by Schmidt *et al.* 2019 [(1)]. The model was built using martini3 lipids, equilibrated, and the molecular dynamics simulation ran using Gromacs as described in Ozturk *et al.* 2024 [(2)]. The martini3 cardiolipin model was derived from Rakesh Vaiwala and K. Ganapathy Ayappa, 2024 [(3)] by converting the SN1 chains from oleic acid to palmitic acid.

Simulation results

Lipid composition	% DOPC	Energy density kJ nm² (each leaflet)	surface area nm²/phosphate
χ = 1.00	100	-431.5	0.588
χ = 0.75	75	-437.2	0.595
χ = 0.50	50	-432.2	0.617
χ = 0.25	25	-429.2	0.639
χ = 0.00	0	-429.7	0.653

Supplementary Data References

1. Schmidt, V., Sidore, M., Bechara, C., Duneau, J.-P. & Sturgis, J. N. The lipid environment of *Escherichia coli* Aquaporin Z. *Biochimica et Biophysica Acta (BBA) - Biomembranes* **1861**, 431–440 (2019).
2. Ozturk, T. N. *et al*. Building complex membranes with Martini 3. *Methods Enzymol* **701**, 237–285 (2024).
3. Vaiwala, R. & Ayappa, K. G. Martini-3 Coarse-Grained Models for the Bacterial Lipopolysaccharide Outer Membrane of Escherichia coli. *J. Chem. Theory Comput.* **20**, 1704–1716 (2024).

Supplementary Movie 1
HS-AFM video of four individual FocA pentamers viewed from the extracellular side. Scan size: 12 nm × 12 nm, Imaging parameter: 0.33 nm per pixel. 1 frame per second.

Supplementary Movie 2
HS-AFM video of lipid membrane fusion process to FocA clusters on mica. Scan size: 125 nm × 125 nm, Imaging parameter: 0.5 nm per pixel. 1 frame per second.

Supplementary Movie 3
HS-AFM video of individual FocA protein cluster dynamics within lipid membranes of varying compositions. Lipid conditions are indicated by the parameter χ in the bottom right corner of each frame. Imaging parameter: 0.5 nm per pixel. 2 frames per second.

Supplementary Movie 4
HS-AFM video of four individual GlpF tetramers viewed from the extracellular side. Scan size: 12 nm × 12 nm, Imaging parameter: 0.33 nm per pixel. 1 frame per second.

Supplementary Movie 5
HS-AFM video of individual GlpF protein cluster dynamics within lipid membranes of varying compositions. Lipid conditions are indicated by the parameter χ in the bottom right corner of each frame. Imaging parameter: 0.5 nm per pixel. 2 frames per second.

Supplementary Movie 6
HS-AFM video of lipid membrane fusion process to GlpF clusters on mica. Scan size: 200 nm × 200 nm, Imaging parameter: 1 nm per pixel. 1 frame per second.

Supplementary Movie 7
HS-AFM video of individual mixed FocA-GlpF cluster dynamics within lipid membranes of varying compositions. Lipid conditions are indicated by the parameter χ in the bottom right corner of each frame. Imaging parameter: 0.5 nm per pixel. 2 frames per second
